# Six‐Month Music Practice Versus Psychomotor Interventions in Mild Cognitive Impairment: Behavioral and Hippocampal Outcomes

**DOI:** 10.1111/nyas.70433

**Published:** 2026-11-02

**Authors:** Clara E. James, Chantal Junker‐Tschopp, Matthias Kliegel, Dimitra Kokkinou, Gilles Allali, Giovanni B. Frisoni, Andrea Brioschi Guevara, Damien Marie

**Affiliations:** ^1^ Geneva Musical Minds Lab, Geneva School of Health Sciences University of Applied Sciences and Arts Western Switzerland HES‐SO Geneva Switzerland; ^2^ Faculty of Psychology and Educational Sciences University of Geneva Geneva Switzerland; ^3^ Geneva School of Social Work, Department of Psychomotricity University of Applied Sciences and Arts Western Switzerland HES‐SO Geneva Switzerland; ^4^ Center for the Interdisciplinary Study of Gerontology and Vulnerability University of Geneva Geneva Switzerland; ^5^ Leenaards Memory Center Lausanne University Hospital and University of Lausanne Lausanne Switzerland; ^6^ University Hospitals and University of Geneva, Memory Center Geneva Switzerland; ^7^ CIBM Center for Biomedical Imaging, Cognitive and Affective Neuroimaging Section University of Geneva Geneva Switzerland

**Keywords:** cognitive performance, hippocampal integrity, magnetic resonance imaging (MRI), mild cognitive impairment (MCI), musical instrument training, psychomotor intervention, sensorimotor performance

## Abstract

Mild cognitive impairment (MCI) is characterized by measurable cognitive decline with largely preserved daily functioning and increased risk of dementia. MCI patients may also exhibit sensorimotor and communication impairments, while hippocampal atrophy and altered functional connectivity are prominent neural features associated with further cognitive decline. Evidence from nonpharmacological interventions integrating cognitive challenge, coordinated movement, and emotional engagement remains limited in early‐stage MCI. In this pilot intervention study, we examined whether 6 months of intensive musical instrument training versus psychomotor interventions were associated with changes in cognitive and sensorimotor performance, hippocampal gray matter volume, and functional connectivity. Outcomes were compared between randomized MCI groups, with matched healthy controls as reference for test−retest and age‐related neural effects. Improvements in global cognition were modest, reaching marginal significance only in the music group, while both interventions were associated with complementary domain‐specific cognitive and sensorimotor gains. Hippocampal gray matter volume remained stable across all three groups, whereas hippocampal functional connectivity declined in healthy controls but remained stable in both intervention groups, consistent with possible functional neural maintenance. These preliminary findings suggest that group‐based, low‐risk music and psychomotor interventions may support cognitive, sensorimotor, and neural functioning in early MCI, warranting further investigation in larger controlled trials.

## Graphical abstract

Six months of music or psychomotor interventions in MCI patients yielded complementary domain‐specific benefits. Music training improved verbal fluency, speech‐in‐noise perception, and short‐term memory, the latter associated with homework duration. Psychomotor intervention enhanced balance, hand dexterity, and body‐schema awareness. Consistent with neural maintenance, hippocampal gray matter and functional connectivity remained stable in both intervention groups, whereas connectivity declined in healthy controls, who were included to assess test‐retest effects and normal brain aging.

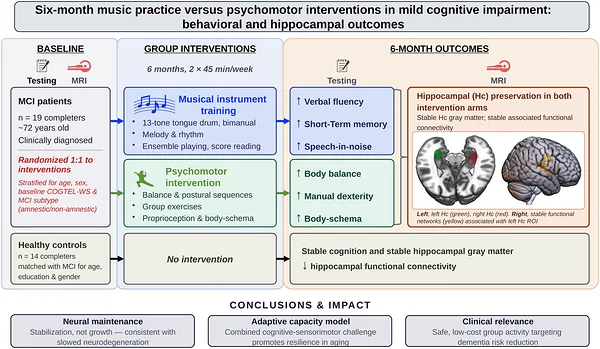

## 1 Introduction

Mild cognitive impairment (MCI) is a heterogeneous clinical condition situated between normal aging and dementia, characterized by measurable cognitive decline without significant impairment in everyday functioning [1−3]. MCI occupies a critical position along the continuum of cognitive aging because it identifies individuals at increased risk for dementia [2]. Beyond core cognitive deficits, individuals with MCI often experience additional impairments in executive function, whole‐body balance, mood, and social communication [4, 5]. Together, these multidomain alterations contribute to reduced quality of life and increased vulnerability. They encompass both sensorimotor disturbances, such as gait and balance impairments [6, 7], as well as auditory–cognitive deficits such as speech‐in‐noise perception [8, 9]. Poorer speech‐in‐noise perception in MCI has been associated with communication difficulties and increased dementia risk [10]. From a cognitive perspective, speech‐in‐noise perception relies on higher‐order auditory cognitive processes, including selective attention, inhibition, working memory, and auditory–motor integration [10].

Recent global epidemiological evidence indicates that the overall prevalence of MCI in the geriatric population is high (∼23.7%). Moreover, reported prevalence has risen over time in association with aging populations worldwide [11], underscoring the growing public health relevance of interventions that may postpone or attenuate neurocognitive decline and reduce the risk of progression to dementia [12]. Effective nonpharmacological interventions that can be integrated into daily living activities are thus of high interest, yet evidence for their impact on MCI remains limited.

Multimodal activities that combine cognitive challenge, emotional engagement, and coordinated movement, such as musical instrument training (hereafter “music training”) or body‐mind interventions, may hold particular promise. The adaptive‐capacity model [13] proposes that combining moderate physical effort with new cognitive challenges can stimulate neuroplasticity and enhance resilience, especially in older adults [13, 14].

Music training serves as a strong example: Playing an instrument in a group setting improved working memory, attention, sensorimotor coordination, and auditory–motor integration, while also providing emotional rewards and social motivation accompanied by structural and functional brain plasticity in healthy older adults [15−20]. Specifically in Alzheimer's disease (AD) and MCI patients, musical activities have been shown to benefit general cognition, executive function, episodic memory, verbal fluency, emotional well‐being, and mood [21−25].

Previous intervention studies provide an important context for the present design. In healthy older adults, Clark et al. [26] reported differential cognitive effects of music training compared to gentle physical activity, highlighting the potential specificity of multimodal auditory–motor engagement. In individuals with MCI, Biasutti and Mangiacotti [27] observed cognitive improvements following approximately 4 months of music‐based interventions compared with soft gymnastics, but these improvements were limited to a subgroup of patients with baseline Mini‐Mental State Examination (MMSE [28]) scores ≤ 23.

More recent randomized controlled longitudinal studies have extended these findings by demonstrating behavioral (e.g., cognitive, sensorimotor, and quality‐of‐life) and neuroplastic changes following two distinct music‐based interventions in healthy older adults [15−17, 29−31].

However, direct comparisons between music‐based and potentially also relevant body‐mind interventions within the same randomized framework remain scarce, particularly in early‐stage MCI and with combined behavioral and neuroimaging outcomes [32].

Complementary to music‐based approaches, psychomotor intervention is a body‐mind approach that has received limited empirical attention in the context of MCI, and targets proprioception, balance, spatial orientation, body representation, and controlled movement, domains often affected in MCI [33]. Group‐based psychomotor interventions in community‐dwelling older adults improved processing speed, executive functions, attention, reaction time, and mobility, with particularly pronounced benefits in lower‐functioning participants [34]. Across community‐dwelling older adults and patients with MCI or AD, psychomotor approaches have been shown to benefit cognition, well‐being, and quality of life, as well as sensorimotor abilities including balance, gait, and mobility [34−40].

Music practice and psychomotor interventions are both ecologically meaningful and well‐suited for older adults, particularly when offered in group settings that promote sustained engagement and social connection in the post‐retirement period [41, 42].

From a neurobiological standpoint, MCI is characterized by vulnerability in specific brain systems. The hippocampus is a key biomarker in MCI, as its atrophy strongly correlates with episodic memory loss and predicts progression to AD [43, 44]. Beyond episodic memory, hippocampal and subfield volumes relate to broader cognitive functions: in a large non‐AD cohort, screened with an MMSE cutoff of <26 and, therefore, likely including individuals with MCI, specific hippocampal subregions were associated with executive functioning and global cognition [45]. In healthy older adults, hippocampal volume was associated with fluid cognition, particularly episodic memory, while left hippocampal volume was also associated with working memory [46]. More pronounced atrophy in amnestic compared to nonamnestic MCI supports a continuum of medial temporal vulnerability across diagnostic groups [47]. Beyond structural degeneration, hippocampal functional alterations in MCI extend into large‐scale network dynamics, with altered hippocampal connectivity to distributed cortical and subcortical regions, including default‐mode network hubs such as the posterior cingulate/precuneus and medial prefrontal cortex [48]. Both hippocampal atrophy and hippocampal hypoconnectivity predict global cognition and nonepisodic memory in MCI [49]. Consequently, hippocampal structure and connectivity provide a sensitive index of multisystem neural decline across the aging AD continuum, and approaches combining hippocampal volumetry with seed‐based whole‐brain connectivity mapping are well aligned with evidence of early hippocampal atrophy and altered large‐scale network connectivity in MCI [48−51]. Together, these findings highlight the hippocampus as relevant for assessing potential intervention‐related changes in MCI. Notably, a recent systematic review of randomized controlled trials on music‐making interventions in older adults reported an absence of studies in individuals with MCI that examined magnetic resonance imaging (MRI) outcomes [32], underscoring a clear gap in the literature and supporting the inclusion of neuroimaging measures in the present study.

The COPE study (Countervail cOgnitive imPairmEnt) was designed as a pilot investigation to examine the potential differential effects of 6 months of music practice versus psychomotor intervention in patients with MCI. A matched healthy observational control group was additionally included to provide a benchmark for behavioral test–retest effects and normal age‐related neuronal decline [52], but not as a comparator for intervention efficacy, because healthy controls (HCs) cannot provide an estimate of the untreated trajectory of MCI. Both interventions were delivered in small groups (3–4 participants) twice weekly for 45 min and included structured daily homework.

The primary outcome was improvement in the COGTEL Weighted Score (COGTEL‐WS) [53], a validated measure of global cognition that covers multiple cognitive domains, shows high correlation with the MMSE (*r* = 0.93), and avoids ceiling effects in healthy older adults. Secondary outcomes included additional cognitive and sensorimotor tests, COGTEL subtests, and structural and functional MRI focusing on hippocampal networks. As a global composite, the COGTEL‐WS represents a conservative outcome measure in a heterogeneous MCI sample; accordingly, domain‐specific cognitive changes and neural maintenance effects were considered particularly informative indicators of intervention efficacy in this pilot trial with a limited sample size [54].

Detecting global cognitive change over short intervention periods is challenging in early‐stage MCI [27]. In addition, complex skill learning, such as focused music practice on a complex instrument, may involve an initial acquisition phase, during which measurable effects remain limited and are not yet reflected in global cognitive composite measures.

These considerations support examining whether multimodal nonpharmacological interventions may enhance or stabilize cognitive and sensorimotor functioning, using conservative general cognitive and domain‐specific effects together with hippocampal volume and network integrity in MCI.

For a broader literature overview, we refer readers to the published protocol [52].

## 2 Materials and Methods

For extensive methodological details of the COPE study (Countervail cOgnitive imPairmEnt), including the rationale for selecting the COGTEL, full hypotheses, comprehensive intervention descriptions, and the broader literature review on which the study was initially grounded and that was updated here, we refer readers to the published protocol [52].

### 2.1 Study Design and Ethics

This randomized controlled trial was registered at ClinicalTrials.gov (NCT04546451) and approved by two Swiss cantonal ethical commissions (Geneva: CCER, and Vaud: CER‐VD). Assessments were performed at baseline (T0), 3 months (T1), and 6 months (T2) post‐intervention.

Importantly, randomization applied only to participants with MCI, who were allocated to the two intervention arms. The HC group was not randomized and served as a matched observational comparison group. Accordingly, the study should be regarded as a 2‐arm randomized controlled trial in MCI participants, supplemented by an external healthy matched control group rather than a conventional 3‐arm RCT. Comparisons involving HCs should, therefore, be interpreted cautiously due to baseline diagnostic differences.

A priori power calculations were reported in the published protocol. Briefly, the analysis was based on the primary cognitive outcome (COGTEL) and informed by Biasutti and Mangiacotti [27], who reported significant cognitive improvement following an approximately 4‐month music‐based intervention in older adults with cognitive impairment (partial *η* ^2^ = 0.296). This effect corresponds to a large Cohen's *f* of 0.65. Using α = 0.05 and a power of 0.80, G*Power 3.1 [55] indicated a required sample size of *n* = 12. Given the strong correlation between MMSE and COGTEL scores (*r* = 0.93) [53], we planned to include 16 participants per group, accounting for an anticipated attrition rate of approximately 20%.

Notably, Biasutti and Mangiacotti [27] further reported that significant cognitive improvements were primarily observed in an MCI subgroup with MMSE scores ≤ 23, which comprised 11 participants in the music intervention group. Despite participant loss related to the COVID‐19 pandemic in the present study, our final sample size, therefore, remains comparable to prior intervention studies reporting significant cognitive effects in cognitively impaired older adults.

The current manuscript reports the interventional longitudinal behavioral and neuroimaging results, that is, changes from baseline to 6 months of intervention. Cross‐sectional comparisons between MCI and HCs at baseline for all variables are published elsewhere [56].

### 2.2 Participants

MCI participants were recruited from the memory centers of the Geneva University Hospitals (HUG) and the Lausanne University Hospital (CHUV).

MCI diagnoses were established at the respective university memory clinics by expert clinicians, in line with European recommendations for memory clinic‐based assessment [12] and contemporary diagnostic workflows integrating clinical, neuropsychological, and neuroimaging evaluation [2], as well as established clinical criteria [3]. Diagnostic procedures included clinical and neurological evaluation, cognitive screening (MMSE and/or Montreal Cognitive Assessment [MoCA] [57]), comprehensive neuropsychological assessment, and exclusion of alternative causes through biological and neuroimaging examinations. Detailed information on the diagnostic procedures, neuropsychological assessment, normative references, and decision criteria used at each center is provided in Supplementary Material 1. The diagnostic framework applied at the CHUV memory center has been previously described [58].

All participants met the inclusion criteria of MMSE ≥ 23 [28] or MoCA ≥ 17 [59, 60]. These thresholds were applied as pragmatic screening criteria within a clinically diagnosed MCI sample, with the aim of excluding individuals with more advanced cognitive impairment or potential early dementia while retaining participants in the early stages of MCI.

Widely used screening thresholds for global cognitive instruments (e.g., MMSE < 24–26; MoCA < 26) are designed to maximize sensitivity for detecting cognitive impairment in general populations [28, 59, 60]. However, distinct cutoffs are often applied in clinical settings to improve specificity and to distinguish MCI from more advanced neurocognitive impairment. In the current study, MCI participants presented no major neurological, psychiatric, or other medical comorbidities, apart from cognitive decline. For detailed inclusion and exclusion criteria, we refer to the published protocol [52]. Attrition and imaging‐related exclusions are summarized in the flow chart in Figure 1.

**FIGURE 1:**
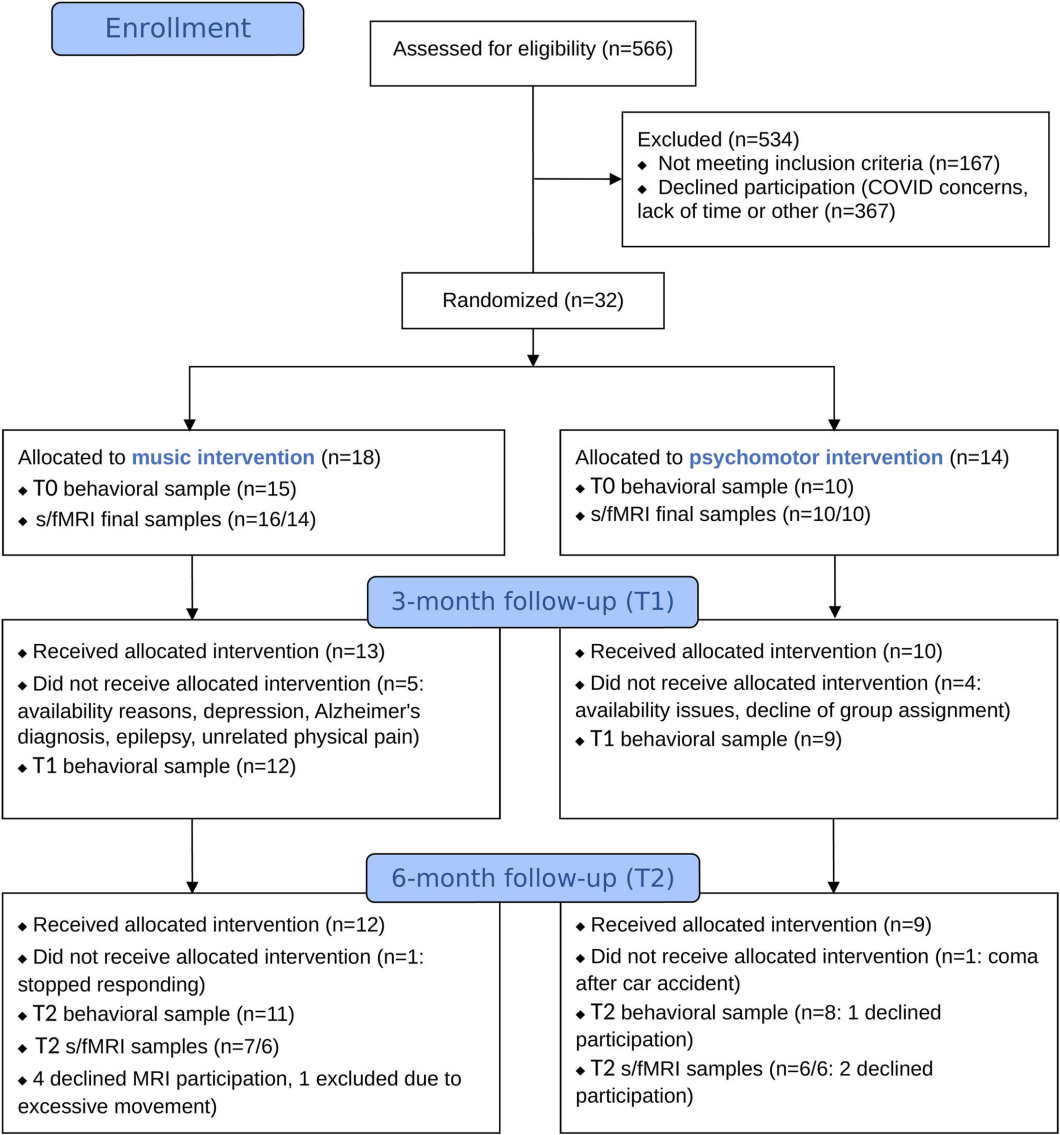
CONSORT flow diagram of participant recruitment, allocation, intervention adherence, follow‐up, and inclusion in MRI analyses across baseline (T0), 3‐month follow‐up (T1), and 6‐month follow‐up (T2). The *n* values are provided separately for the structural and functional MRI samples. See the main text for full information. Abbreviations: MRI, magnetic resonance imaging; s/fMRI, structural/functional MRI.

The control group of healthy older adults (HC) was matched to the MCI group for age, sex, and education level. They were recruited in the Geneva area among community‐dwelling older adults through local advertisements and online platforms, after completion of the experimental group recruitment, to allow group matching. The mean COGTEL‐WS of the HC group at baseline (31.9 ± 7.8) exceeded the mean reported for cognitively healthy older adults in the original instrument validation study (27.98 ± 8.64; *N* = 83 [61]), confirming normal cognitive functioning in our HC sample (Table 1).

**TABLE 1:** Participant characteristics for HC and MCI groups at baseline (T0) and after the 6‐month interventions (T2).

Mean ± standard deviation/number (percentage)			Group comparison	
T0 matching factors for baseline population	HC (*N* = 15)	MCI (*N* = 26)	Statistic	*p*‐value
Age	70.3 ± 4.6	71.5 ± 7.5	*W* = 158.5	0.32
Gender	13 ♀ (87%)	18 ♀ (69%)	*χ* ^2^ = 1.6	0.21
Education level	3.7 ± 1.3	3.2 ± 1.4	*χ* ^2^ = 2.9	0.58
COGTEL‐WS	31.9 ± 7.8	22.0 ± 7.5	*t* = −4.0	< 0.001
**T0 randomization factors for baseline MCI subsample**	**MUS (*N* = 18)**	**PM (*N* = 14)**		
Age	72.1 ± 7.4	69.9 ± 7.2	*W* = 144.5	0.49
Gender	10 ♀ (56%)	10 ♀ (71%)	*χ* ^2^ = 0.85	0.36
COGTEL‐WS	22.4 ± 8.4	21.4 ± 6.0	*t* = 0.8	0.40
Amnestic patients	4 (22%)	5 (36%)	*χ* ^2^ = 0.79	0.40
**T0 matching factors for longitudinal population**	**HC (*N* = 14)**	**MCI (*N* = 19)**		
Age	70.4 ± 4.7	72.6 ± 7.3	*t* = 1.0	0.33
Gender	12♀ (86%)	13♀ (68%)	*χ* ^2^ = 1.3	0.25
Education level	3.6 ± 1.3	3.1 ± 2.1	*χ* ^2^ = 3.0	0.56
COGTEL‐WS	31.7 ± 8.1	22.6 ± 8.1	*t* = −3.2	0.003
**T0 randomization factors for longitudinal MCI subsample**	**MUS (*N* = 11)**	**PM (*N* = 8)**		
Age	73.2 ± 7.1	71.9 ± 8.0	*W* = 46	0.93
Gender	7♀ (63.7%)	6♀ (75%)	*χ* ^2^ = 0.28	0.60
COGTEL‐WS	25 ± 9.6	19.4 ± 4.1	*t* = 1.53	0.14
Amnestic patients	3 (27%)	5 (63%)	*χ* ^2^ = 2.4	0.12

*Note*: The HC and MCI groups did not differ significantly in age, sex distribution, or years of education. Within the MCI cohort, the two intervention conditions (MUS and PM) likewise showed no significant differences in age, sex, COGTEL‐WS, or the proportion of amnestic versus nonamnestic subtypes. COGTEL‐WS differed significantly between HC and MCI.

Abbreviations: COGTEL‐WS, COGTEL weighted score; HCs, healthy controls; MCI, mild cognitive impairment; MUS, music intervention; PM, psychomotor intervention; *t*, Student's *t*‐test statistic; W, Wilcoxon rank‐sum statistic; ♀, female; χ^2^, chi‐square statistic.

In contrast, the mean COGTEL score of the MCI group (22.0 ± 7.5) closely matched the cutoff for mild neurocognitive disorder (21.75) [62], supporting the clinical MCI diagnosis and confirming that our participants represented a relatively high‐functioning MCI group. Moreover, the COGTEL‐WS was significantly lower in the MCI group than in the matched HC group (22.0 ± 7.5 vs. 31.9 ± 7.8, *p* < 0.001; Table 1).

Of the 32 randomized participants included, 21 completed the post‐intervention behavioral assessment at T1 and 19 completed the follow‐up behavioral assessment at T2. At baseline (T0), 16 participants in the music intervention group (MUS) underwent structural MRI (sMRI) and 14 functional MRI (fMRI); in the psychomotor group (PM), 10 participants underwent both structural and functional MRI. At T2, usable sMRI data were available for 13 participants (MUS: *n* = 7; PM: *n* = 6) and usable fMRI data for 12 participants (MUS: *n* = 6; PM: *n* = 6). Attrition at T1 was due to availability constraints, depression, Alzheimer's disease/epilepsy diagnosis, unrelated physical pain, and decline of group assignment, whereas attrition at T2 resulted from refusal to undergo repeated MRI because of fatigue (*n* = 6), excessive movement during MRI acquisition, and coma following a car accident.

### 2.3 Randomization

Randomization used stratified automated clustering to balance age, sex, baseline COGTEL‐WS, and MCI subtype (amnestic/nonamnestic), as determined by specialists at the university memory centers [52] (see Supplementary Material 1 for details).

### 2.4 Interventions

Both interventions took place in small groups (∼3−4 participants) twice weekly for 45 min over 6 months, complemented by structured daily homework (recommended ∼20 min per day, five times per week), monitored via weekly self‐reported practice‐time forms.

#### 2.4.1 Music Intervention (MUS)

Participants learned to play a 13‐tone steel tongue drum. Progressive training evolved from bimanual rhythmic‐melodic patterns to playing entire melodies, score reading, and ensemble playing. These tasks require sustained and focused attention, executive control, working memory, and higher‐order auditory processing. Daily homework included guided practice of musical material acquired during the sessions, supported by sheet music presented in graded levels of musical notation, ranging from simplified to traditional notation. The teacher held a master's degree in music performance and possessed experience working with individuals with disabilities. Lessons, materials (including a personal tongue drum), and transportation were provided free of charge. A more detailed description of the music intervention is available in the published protocol [52] and in Supplementary Material 2.1.

#### 2.4.2 Psychomotor Intervention (PM)

This intervention focused on integrating motor, sensory, emotional, and cognitive processes through body‐based activities, aiming to support sensorimotor–cognitive integration, body schema awareness, and emotion regulation. Sessions included grounding exercises, guided self‐massage, slow postural transitions, guided movement sequences, and dyadic coordination promoting sensorimotor–emotional attunement. Meaningful choreographed sequences (“the phoenix” [a mythical bird], “the earth,” etc.) were progressively learned and memorized. An instruction booklet with photographs of the sequences supported home practice. The instructor held a master's degree in psychomotor therapy and had experience working with individuals with disabilities. Lessons, materials, and transportation were provided free of charge. A more detailed description of the psychomotor intervention is available in the published protocol [52] and in Supplementary Material 2.2.

### 2.5 Healthy Controls

Fourteen HCs completed the same assessments but received no intervention. They were financially compensated.

### 2.6 Compensation

HCs were compensated at CHF 25/h, whereas MCI participants received twice‐weekly interventions free of charge, with travel costs reimbursed and materials provided (a personal tongue drum was loaned for the duration of the study, while sheet music and instruction booklets were provided).

### 2.7 Behavioral Data Collection

Behavioral data were collected using a cognitive‐sensorimotor battery selected for sensitivity to MCI symptoms [52]. Tests covered multiple domains and were pseudorandomized across participants and time points. The clock‐drawing test [63] was excluded because it is included in the MoCA administered during the MCI diagnostic procedure at the CHUV, potentially introducing test–retest effects.

#### 2.7.1 Primary Outcome

General cognition was assessed with the Cognitive Telephone Screening Instrument (COGTEL) [53, 61], administered face‐to‐face. The COGTEL provides subtest scores for verbal short‐term memory, verbal long‐term memory, working memory (backward digit span), verbal fluency, inductive reasoning, and prospective memory, as well as a weighted composite score. This weighted composite score (COGTEL‐WS) was the primary outcome of the study.

#### 2.7.2 Secondary Outcomes

Speech‐in‐noise (SIN) perception was measured with the International Matrix Test [29, 64, 65]; the binaural speech reception threshold (SRT), expressed in dB SNR, was sign‐inverted so that higher scores reflected better performance. Participants with poor speech intelligibility (speech without noise <80%) were excluded [66].

The Trail Making Test indexed processing speed (TMT‐A) and cognitive flexibility (TMT‐B) [67]; completion times were sign‐inverted so that higher scores reflected better performance.

Selective and sustained attention was assessed using the d2‐R, with performance quantified by the Concentration Index (CI = number of perceived targets − [number of omission errors + confusion errors]) [68, 69]. The term “d2” refers to the target stimulus, namely, the letter “d” accompanied by two strokes.

Manual dexterity and bimanual coordination were assessed with the Purdue Pegboard Test (PPT), using scores for the right hand, left hand, both hands, and assembly subtests, as well as their sum, referred to as the total score (PPT‐TS) [70].

Left/right foot static balance (hereafter “balance”) was measured using the Unipedal Balance Test (UBT) [71].

Motor inhibition was quantified using the Go/NoGo d‐prime score (d′) [72], calculated as d′ = *z*(Go hits) − *z*(NoGo false alarms), where *z* denotes the *z*‐score transformation of hit and false‐alarm rates.

Body‐schema awareness was evaluated through hand and foot laterality recognition [73].

COGTEL subtests included verbal short‐term memory, verbal long‐term memory, working memory (backward digit span), verbal fluency, inductive reasoning, and prospective memory. The COGTEL subtest of verbal fluency comprises letter and category fluency tasks that engage executive functions involved in controlled and strategic lexical retrieval [53].

Behavioral assessments were administered by trained assessors with a master's degree in psychology, following standardized procedures. Assessors were not formally blinded to group allocation. However, all testing followed structured protocols to minimize bias.

All participants included in the analyses were able to complete the full assessment battery. Dropout was unrelated to the psychometric testing procedures.

The measures used are standardized and widely validated instruments with established psychometric properties reported in the literature.

#### 2.7.3 Participant Characteristics

Table 1 lists the variables used to describe randomization across intervention groups, matching between MCI and HC, and participant retention. After testing normality for continuous variables (age, COGTEL‐WS), group comparisons were conducted using Student's *t*‐tests or Mann−Whitney *U* tests. Categorical variables (education, gender, MCI subtype) were evaluated with χ^2^ tests.

#### 2.7.4 Behavioral Data Analyses

For all behavioral outcomes, change scores were computed by subtracting baseline from follow‐up performance (T1–T0, T2–T0) to directly quantify within‐subject change over time and facilitate interpretation of training‐related effects. Because 3‐month analyses did not yield significant effects, subsequent analyses focused on 6‐month change scores (T2–T0). Intervention group effects on the primary outcome (COGTEL‐WS) were examined using ANCOVAs, whereas COGTEL subtests and other cognitive and sensorimotor measures were analyzed using linear mixed‐effects models (LMMs).

Three series of comparisons were conducted: (1) intervention effects on the primary outcome (COGTEL‐WS); (2) broader cognitive and sensorimotor changes across HC and MCI groups, combining MUS and PM participants; and (3) pairwise comparisons between the active intervention groups (MUS vs. PM). Follow‐up comparisons were performed when omnibus effects were significant. In addition, selected within‐group changes over time (T2−T0) in each MCI subgroup (MUS and PM) were examined using one‐sample *t*‐tests based on a priori hypotheses. All models included Age and Site as covariates, with Subject entered as a random effect.

Analyses were conducted on participants with available data at the relevant time points. Because change scores required complete observations, analyses followed an available‐case (completers) approach rather than a strict intention‐to‐treat framework. No imputation of missing data was performed.

#### 2.7.5 COGTEL‐WS (Primary Outcome)

An ANCOVA tested group effects (HC, MUS, PM) on 6‐month changes in the COGTEL‐WS (T2−T0).

#### 2.7.6 HC Versus MCI: COGTEL Subtests, and Broader Cognitive and Sensorimotor Domains

For COGTEL subtests, *z*‐scored T2−T0 changes were entered into an LMM with Subtest and Group (HC, MCI) as fixed factors. Prospective‐memory changes (−1/0/+1) were tested using χ^2^.

To compare 6‐month change across cognitive and sensorimotor domains (combined cognitive‐sensorimotor battery), another LMM included Group and Test (10 measures: ‐SRT, ‐TMT‐A/B, d2‐CI, hand/foot laterality, PPT‐TS, left/right UBT, Go/NoGo d’). Furthermore, a separate model examined the four Purdue Pegboard subtests (left hand, right hand, both hands, and assembly).

#### 2.7.7 MUS Versus PM: Cognitive and Sensorimotor Outcomes

Two LMMs assessed 6‐month changes comparing MUS (*n* = 11) to PM (*n* = 8) across the 10 psychometric tests and six COGTEL subtests: *z*(T2−T0 performance) ∼ Group × Psychometric Measure + Site + Age + (1 | Subject). A subsequent exploratory, parsimonious LMM based on the same statistical model targeted three measures: two COGTEL subtests (categorical fluency and verbal short‐term memory) and one laterality measure (foot laterality).

We selected this parsimonious set of variables partly based on our a priori hypotheses, as described in the published study protocol ([52], pp. 5–6), and partly on visual inspection of the LMM plots. Specifically, we hypothesized greater cognitive improvements in the music group (COGTEL‐WS and subtests) and greater sensorimotor improvements in the psychomotor group, particularly in laterality measures assessing body‐schema awareness. However, the three measures included in the parsimonious model were not individually prespecified and were partly selected based on the observed data. Accordingly, this analysis should be considered exploratory. Comparisons within this parsimonious set were adjusted for multiple testing using the Holm procedure.

Partial correlations (controlling for Age and Site) examined whether homework duration was associated with training benefits: categorical fluency and verbal short‐term memory in MUS, and foot laterality in PM. Normality of the variables was verified using Shapiro−Wilk tests.

#### 2.7.8 Within‐Group MCI Outcomes

Based on prior work [29, 38, 65, 74], one‐sample *t*‐tests (after normality checks) evaluated 6‐month changes in motor and SIN outcomes within each MCI intervention group.

### 2.8 MRI Data Acquisition and Preprocessing

MRI data were acquired on 3T Siemens Prisma Fit scanners (Siemens, Erlangen, Germany) at both sites (Geneva: CMU Brain and Behaviour Laboratory, Centre Médical Universitaire; Lausanne: LREN, Laboratoire de Recherche en Neuroimagerie, CHUV, Centre Hospitalier Universitaire Vaudois).

#### 2.8.1 Structural MRI and Voxel‐Based Morphometry

High‐resolution T1‐weighted images were acquired with an MP2RAGE sequence (1 mm^3^; 176 slices; TR/TE = 5000/2.98 ms; TI1/TI2 = 700/2500 ms; 8:22 min). Preprocessing and analyses used MATLAB R2023a, SPM12 [75], and CAT12 [76, 77], following an advanced voxel‐based morphometry (VBM) workflow [78].

At baseline, images were denoised, segmented into gray matter (GM), white matter (WM), and cerebrospinal fluid (CSF), and total intracranial volume (TIV) was estimated. Segmented images were then normalized to MNI space using DARTEL [79, 80], modulated, and smoothed with an 8‐mm full width at half maximum (FWHM) Gaussian kernel. Image quality was assessed using CAT12 quality‐assurance metrics.

Longitudinal VBM followed CAT12's dedicated pipeline, which jointly registered T0 and T2 images to preserve intraindividual consistency. Images underwent inverse‐consistent realignment, bias‐field correction, DARTEL normalization, Jacobian modulation, and 8‐mm FWHM smoothing, with additional homogeneity and lesion checks.

#### 2.8.2 Resting‐State fMRI Preprocessing

Resting‐state fMRI was acquired using a multiband EPI sequence (2.5 mm^3^ isotropic; TR/TE = 1350/31.6 ms; acquisition time: 10:31 min). Participants were instructed to fixate on a central cross on the computer screen. Preprocessing was performed in CONN v22.a [81] in SPM12, and included motion‐corrected realignment, slice‐timing correction, outlier detection, tissue segmentation, and normalization to MNI space. Images were subsequently smoothed with a 5‐mm FWHM Gaussian kernel.

Motion artifacts were regressed using the Friston 24‐parameter model [82], and nuisance regressors (WM, CSF, global signal) were removed. Data were normalized with DARTEL [79, 80], and band‐pass filtered (0.008−0.09 Hz).

### 2.9 MRI Data Analyses

#### 2.9.1 Definition of Regions of Interest

Because this study focused on longitudinal intervention effects and the T2 MRI sample was modest, analyses were restricted to bilateral hippocampal regions of interest (ROIs), consistent with the a priori target region motivated in the Introduction. Population‐based hippocampal ROIs (Figure 2) were derived from a baseline VBM contrast MCI < HC, identifying voxels where HCs showed greater GM volume than individuals with MCI (*p* < 0.05, family‐wise error corrected [FWE‐corr]), cf. Ref. [56]. These significant left and right hippocampal clusters, reflecting regions of reduced GM volume in MCI, were binarized and used as masks to extract individual GM volume at T0 and T2 using SPM's get_totals function. This approach allowed us to examine intervention effects in hippocampal regions statistically defined as vulnerable in MCI, both in terms of GM integrity and functional connectivity.

**FIGURE 2:**
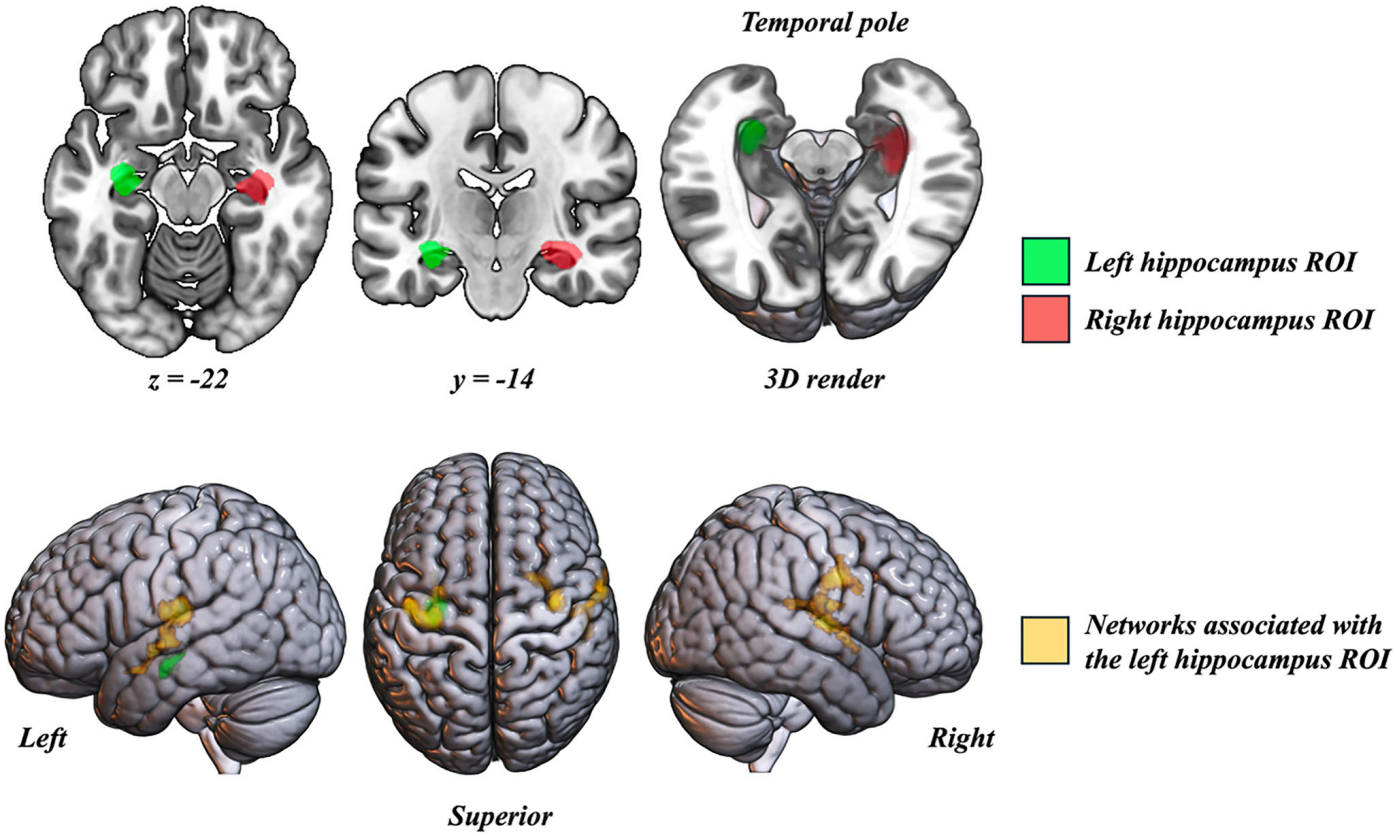
Hippocampal regions showing reduced gray matter volume in MCI at baseline (T0), defined using the MCI < HC contrast (*p* < 0.05 FWE‐corr), and used as ROIs for gray matter volume extraction (green = left; red = right). The lower panels depict the functional networks associated with the left hippocampal ROI (yellow) used for connectivity analyses (seed regions in green). Abbreviations: HCs, healthy controls; MCI, mild cognitive impairment.

While this ROI definition is partly data‐driven, it was constrained by the limited sample size at follow‐up and the resulting lack of statistical power for whole‐brain longitudinal analyses. The hippocampus was selected as an a priori region of interest based on its well‐established role as a core biomarker in MCI and Alzheimer's disease (see Introduction).

Importantly, ROIs were derived from baseline group differences (MCI < HC) to ensure that subsequent longitudinal analyses were anchored in empirically defined, disease‐relevant regions showing structural vulnerability in MCI. This approach allowed us to focus on MCI‐sensitive hippocampal subregions, rather than relying on anatomical templates that may include structurally preserved voxels not representative of disease‐related vulnerability. Such an approach is consistent with evidence linking atrophy‐defined regions to large‐scale functional network alterations in MCI [48, 83].

We acknowledge that defining ROIs based on group differences may introduce bias and limit generalizability. Accordingly, results are interpreted as region‐specific effects within MCI‐vulnerable hippocampal areas and should be considered hypothesis‐generating in the context of this pilot study.

#### 2.9.2 Longitudinal Structural GM Analysis

Two LMMs were fitted separately for each hemisphere. Mean GM hippocampal volume served as the dependent variable; Subject was modeled as a random effect. Fixed factors were Time (T0 vs. T2), Group (MUS, PM, HC), and their interaction, with Age, Site, and TIV as covariates, like in the previous analyses.

#### 2.9.3 Longitudinal Seed‐Based Functional Connectivity Analysis

Seed‐based functional connectivity analyses were performed at baseline using the left hippocampal ROI. The baseline contrast MCI < HC (*p* < 0.005, uncorrected at the voxel level; *p* < 0.05, false discovery rate [FDR]‐corrected at the cluster level) identified bilateral sensorimotor and executive‐control networks connected to the left hippocampus [56]. Individual Fisher‐transformed connectivity values for these networks (networks of interest; Figure 2) were extracted at T0 and T2. Connectivity changes were analyzed using the same LMM structure with Time, Group, and their interaction as fixed effects, Age, Site, and TIV as covariates, and Subject as a random factor.

This strategy ensures that longitudinal connectivity analyses track changes within functionally relevant networks anchored in structurally compromised hippocampal regions, thereby increasing sensitivity to intervention‐related effects in a pilot sample.

### 2.10 Statistical Analyses of Behavioral Variables

To characterize between‐group differences, effect sizes are reported as Hedges’ *g* for pairwise comparisons and partial *η* ^2^ for multi‐df omnibus *F*‐tests. Hedges’ *g* is a standardized effect size measure that corrects for small‐sample bias [84], derived from Cohen's *d* using the following formulas: *d* = estimate / SD_effect; SD_effect = (√*N*) × standard error; *g* = *d* × (1 – (3 / (4(*N* – 2) – 1)).

### 2.11 Software Used for Statistical Analyses of Behavioral and Brain Variables

Statistical analyses were performed using JASP (0.18.2, Apple Silicon). Age and Site (1: Geneva, 2: Lausanne) were added as covariates in all statistical analyses. Additionally, analyses involving GM volume were also controlled for TIV.

## 3 Results

Participant characteristics are provided in Table 1. Fifteen HC and 26 MCI participants started the study. At T2, 14 HC, 11 MUS, and eight PM completed the measurements, for a total of 33 participants who completed the 6‐month follow‐up (see Figure 1).

### 3.1 Course Attendance and Homework

Participants in the MUS group (*n* = 11) attended a mean of 35.9 ± 2.5 (SD) sessions out of the 40 scheduled sessions over 6 months, compared with a mean of 34.3 ± 3.0 (SD) sessions in the PM group; the difference between the groups was not significant (*t*(17) = 1.31, *p* = 0.21, Hedges’ *g* = 0.58). Over the 6‐month intervention period, comprising 40 interventions in total (twice per week, except holidays), the total time spent on homework was 2680 ± 1547 min in the MUS group and 2075 ± 1178 min (34.6 ± 19.6 h) in the PM group. This corresponded to an average of approximately 134 min (2.2 h) per intervention week in the MUS group and 104 min (1.7 h) per intervention week in the PM group. Homework duration did not differ significantly between groups either (*t*(17) = 0.93, *p* = 0.37, Hedges’ *g* = 0.41).

### 3.2 Safety

As required by the local ethics committee, study‐related adverse events were monitored; none were reported.

### 3.3 Behavioral Results

For interpretation, change scores are expressed as T1−T0 or T2−T0, such that positive, near‐zero, and negative *z*‐score changes indicate improvement, stability, and decline, respectively.

### 3.4 Primary Outcome

#### 3.4.1 Three‐Month Longitudinal Behavioral Analyses (T1−T0)

Across the first 3 months of interventions, no significant group effects (HC vs. MCI, MUS vs. PM) emerged at the *p* < 0.05 threshold for changes in COGTEL‐WS scores.

#### 3.4.2 Six‐Month Longitudinal Behavioral Analyses (T2−T0)

MUS yielded a marginal improvement in COGTEL‐WS from T0 to T2 (estimated mean change = 4.2 ± 2.1; *t* = 2; *p* = 0.06, Hedges’ *g* = 0.54). No significant gains were detected in the PM group (2.6 ± 2.5; *p* = 0.30) or among HC (3.8 ± 2.7; *p* = 0.17).

### 3.5 Secondary Outcomes

#### 3.5.1 Three‐Month Longitudinal Behavioral Analyses (T1−T0)

No significant between‐group differences (HC vs. MCI, MUS vs. PM) were observed from T0 to T1 in any COGTEL subtest or in the additional cognitive or sensorimotor outcomes across the three groups.

#### 3.5.2 Six‐Month Longitudinal Behavioral Analyses (T2−T0)

##### 3.5.2.1 HC Versus MCI: 6‐Month Change (T2−T0) in COGTEL Subtests

Across the seven COGTEL subtests, changes from baseline to 6 months (T2−T0) did not differ significantly between HC and MCI participants, as indicated by a nonsignificant main effect of Group (*F*(1, 29) = 0.02, *p* = 0.89, Hedges’ *g* = 0.02). COGTEL subtest × Group interaction was not significant either (*F*(5, 155) = 1.05, *p* = 0.39, partial *η* ^2^ = 0.03). A significant negative correlation with age was observed across groups (ρ = –0.17, *p* < 0.02).

##### 3.5.2.2 HC Versus MCI: 6‐Month Change (T2−T0) in Multidomain Cognitive and Sensorimotor Performance

For the combined cognitive‐sensorimotor battery, a modest yet significant main effect of Group emerged (*F*(1, 28.40) = 4.2, *p* < 0.05, Hedges’ *g* = –0.348) (Figure 3), whereas the Group × Test interaction was not significant (*F*(9, 263.44) = 1.14, *p* = 0.34, partial *η* ^2^ = 0.04). Overall, MCI participants showed greater multidomain improvement than HC (HC‐MCI estimated marginal mean comparison = −0.35, SE = 0.17, *p* = 0.04).

**FIGURE 3:**
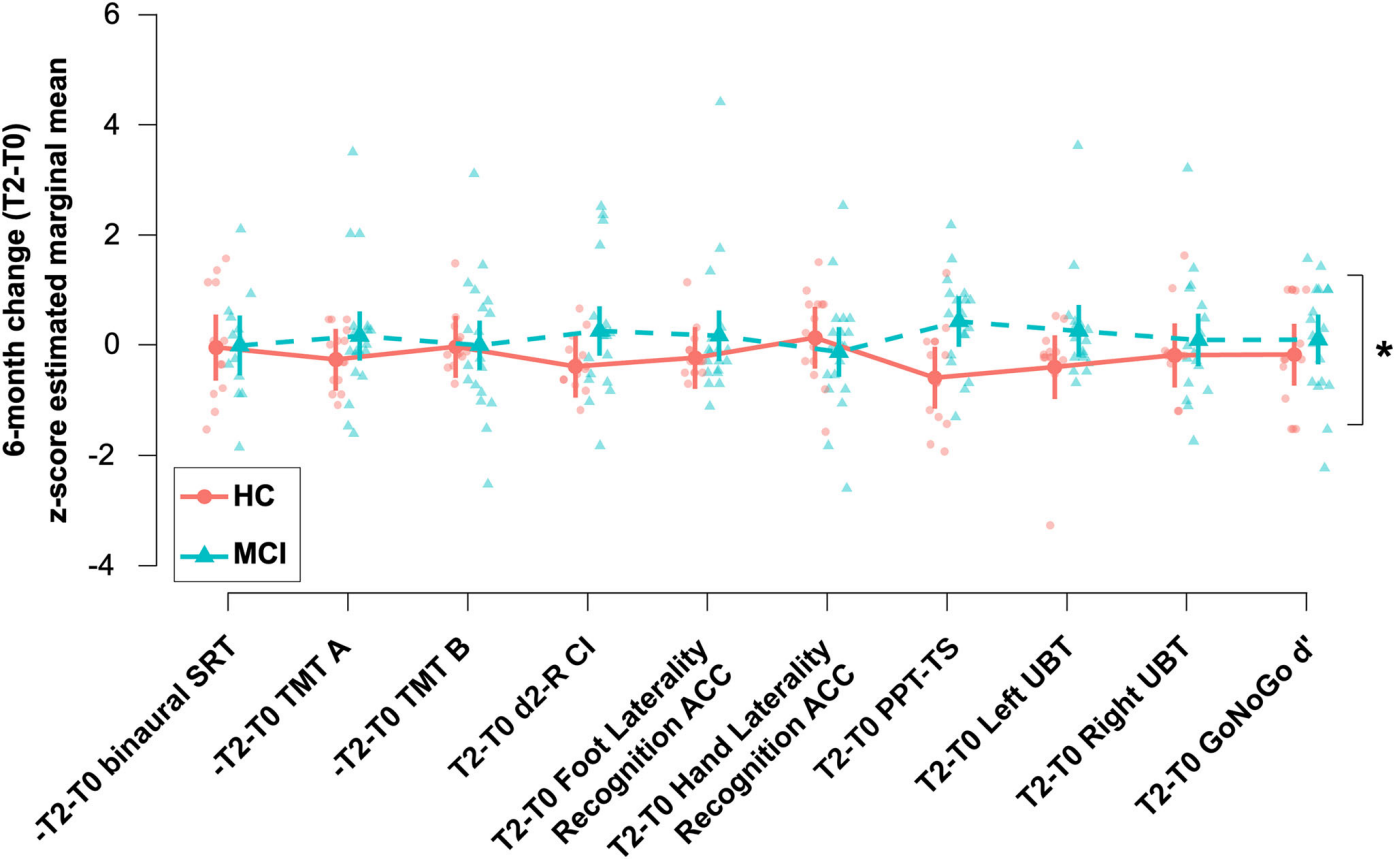
Estimated marginal mean changes over 6 months (T2−T0 *z*‐scores) for each psychometric measure in HC and MCI participants. For binaural SRT, TMT A, and TMT B, raw scores were sign‐inverted prior to *z*‐scoring so that, as for all other measures, positive values reflect better performance. The asterisk indicates the significant group effect on overall change. HCs are shown in red and MCI in cyan. Abbreviations: CI, Concentration Index of the d2‐R; d′, d‐prime of GoNoGo; HCs, healthy controls; MCI, mild cognitive impairment; PPT‐TS, Purdue Pegboard Test Total Score; SRT, speech reception threshold; TMT, Trail Making Test; UBT, Unipedal Balance Test.

Concerning within‐group changes, the MCI arm demonstrated a modest positive change (estimated marginal mean = 0.13, SE = 0.09, *p* = 0.15) and the HC arm showed a slight decline (–0.22, SE = 0.14; *p* = 0.12) across T2−T0; no within‐group change reached statistical significance.

Notably, before the interventions, eight of these tests showed significant differences in favor of the HC group, as reported in a separate publication comparing baseline data from HC and MCI participants from the same cohort (Figure 1A of [56]). Thus, the present findings indicate a change in the overall multidomain pattern, in favor of MCI participants who completed the interventions.

##### 3.5.2.3 HC Versus MCI: 6‐Month Change (T2−T0) in Purdue Pegboard Subtests Performance

A complementary LMM on individual Purdue Pegboard subtests revealed a significant overall Group effect (*F*(1, 28) = 10.6, *p* < 0.005, Hedges’ *g* = 1.11) (Figure 4 and Table 2). Across subtests, the MCI group showed relative stability or slight improvement over 6 months, reflected in positive or near‐zero *z*‐score changes, whereas the HC group showed negative z‐score changes, indicating performance declines. The group difference was significant across all subtests except the left‐hand task.

**FIGURE 4:**
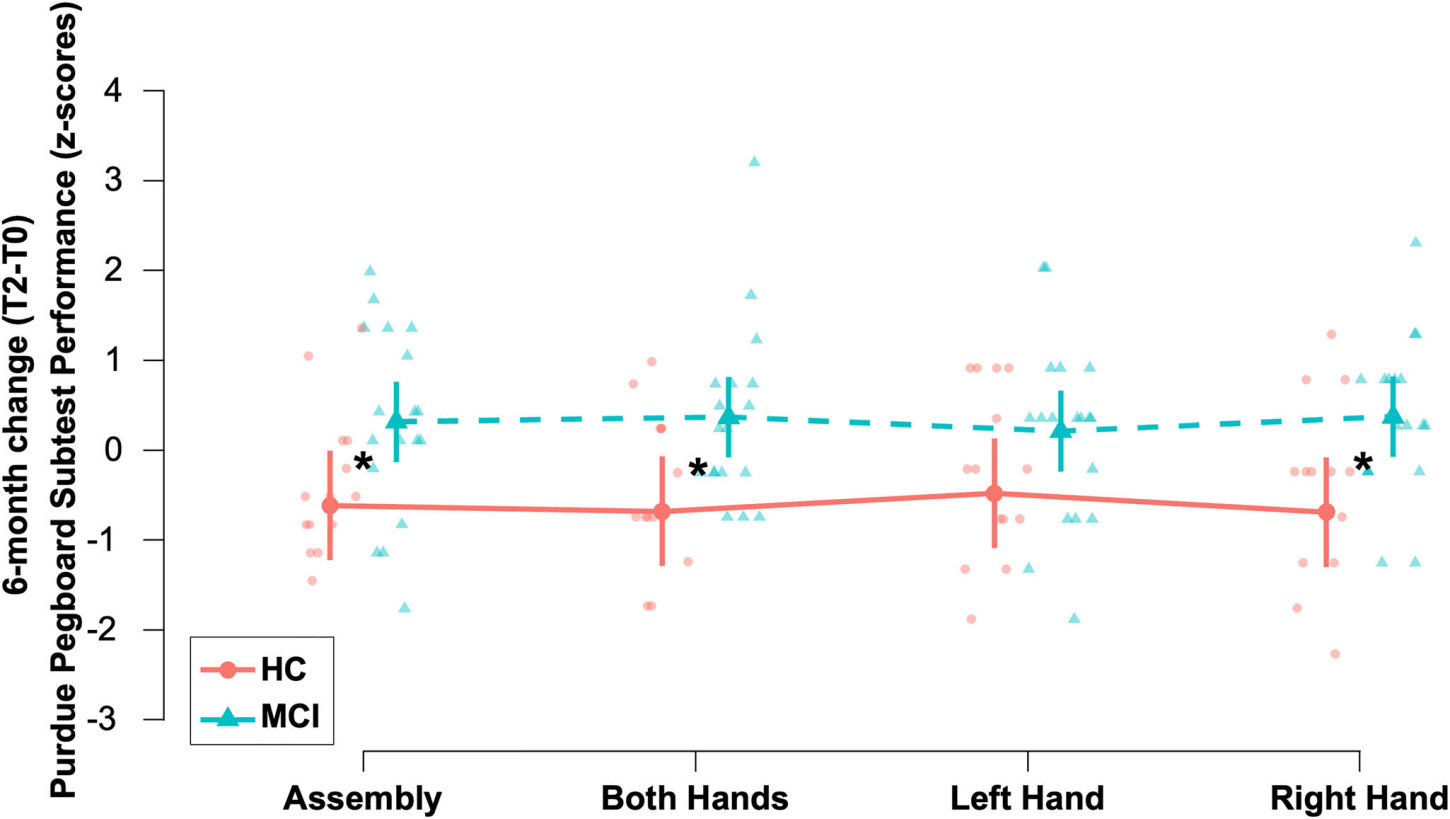
Estimated marginal mean changes over 6 months (T2−T0 *z*‐scores) for each Purdue Pegboard subtest in HC (in red) and MCI (in cyan). HC participants showed negative *z*‐score changes (declines) across most subtests, whereas MCI participants exhibited relative stability or slight improvement, with z‐scores near or above zero. Between‐group differences were significant for all subtests except the left‐hand task. Asterisks indicate significant between‐group differences. Abbreviations: HCs, healthy controls; MCI, mild cognitive impairment.

**TABLE 2:** Estimated marginal mean comparisons between HC and MCI for 6‐month changes (T2−T0) in Purdue Pegboard subtest *z*‐scores.

T2−T0 HC‐MCI comparison	Estimate	SE	95% CI (lower)	95% CI (upper)	*z*	*p*_Holm_	Hedges’ *g*
*z*(Right Hand)	−1.060	0.383	−1.811	−0.309	−2.766	0.0227*	−0.470
*z*(Both Hands)	−1.046	0.383	−1.798	−0.295	−2.730	0.0227*	−0.464
*z*(Assembly)	−0.931	0.383	−1.683	−0.180	−2.430	0.0302*	−0.413
*z*(Left Hand)	−0.692	0.383	−1.443	0.059	−1.806	0.0710	−0.307

*Note*: All subtests showed significant HC‐MCI differences except the left‐hand subtest. Results are ordered by decreasing effect size (Hedges’ *g*). Holm‐corrected significant *p*‐values are indicated with an asterisk.

Abbreviations: CI, confidence interval; HCs, healthy controls; MCI, mild cognitive impairment; SE, standard error.

Although none of the MCI within‐group changes remained significant after Holm‐corrected comparisons, HC participants exhibited significant declines in three subtests: Assembly (estimated marginal mean = −0.62, SE = 0.31, *z* = −2.02, *p*_Holm_ = 0.043), Both Hands (mean = −0.68, SE = 0.31, *z* = −2.23, *p*_Holm_ = 0.026), and Right Hand (mean = −0.69, SE = 0.22, *z* = −2.26, *p*_Holm_ = 0.024). Taken together, these findings indicate relative stability in manual dexterity among MCI participants and marked deterioration among HC participants.

#### 3.5.3 Within MCI: MUS Versus PM 6‐Month Change (T2−T0) for COGTEL Subtests and Foot Laterality Recognition

Analysis of 6‐month changes (T2−T0 *z*‐scores) in COGTEL Categorical Fluency, COGTEL Verbal Short‐Term Memory, and Foot Laterality Recognition Accuracy (“parsimonious LMM”) revealed a significant Psychometric Measure × Group interaction (*F*(2, 33.64) = 10.1, *p* < 0.0004, partial *η* ^2^ = 0.38) (Figure 5 and Table 3).

**FIGURE 5:**
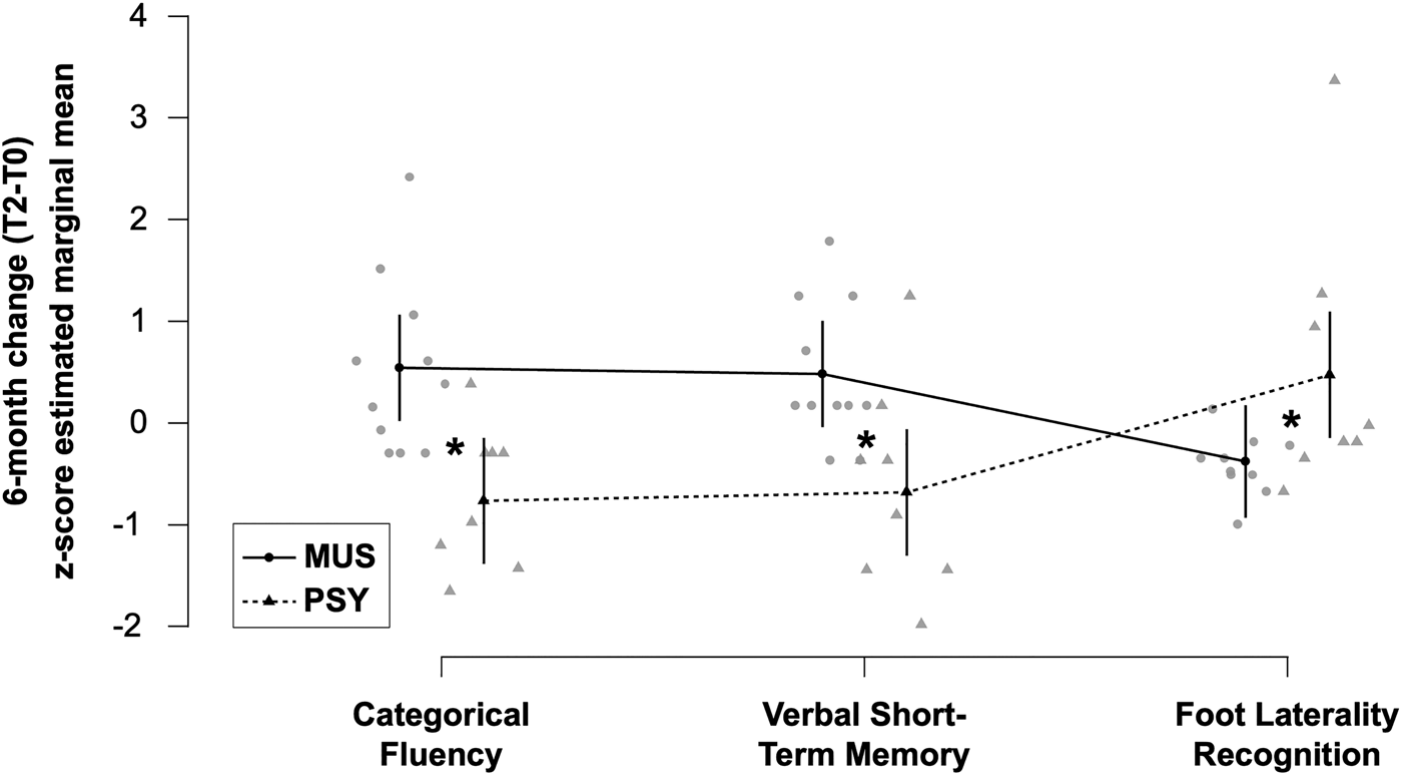
Plot of estimated marginal mean *z*‐score changes (T2−T0) for the COGTEL subtests Categorical Fluency and Verbal Short‐Term Memory, and for Foot Laterality Recognition in the MUS and PM groups. The MUS group exhibited greater change in Categorical Fluency and Verbal Short‐Term Memory, whereas the PM group showed greater change in Foot Laterality Recognition. Asterisks denote significant between‐group differences. Abbreviations: MUS, music intervention; PM, psychomotor intervention.

**TABLE 3:** Comparisons of estimated marginal mean *z*‐score changes (T2–T0) between the MUS and PM groups for the COGTEL subtests Categorical Fluency and Verbal Short‐Term Memory, and for Foot Laterality Recognition.

T2−T0 MUS‐PM comparison	Estimate	SE	95% CI (lower)	95% CI (upper)	*z*	*p*_Holm_	Hedges’ *g*
*z*(Categorical Fluency)	1.308	0.406	0.512	2.104	3.221	0.0038*	0.710
*z*(Verbal Short‐Term Memory)	1.163	0.406	0.367	1.959	2.864	0.0084*	0.630
*z*(Foot Laterality Recognition)	−0.850	0.416	−1.665	−0.035	−2.043	0.0410*	−0.450

*Note*: Effects are ordered by decreasing Hedges’ *g*. Holm‐adjusted comparisons indicated significantly greater changes in the MUS group for Categorical Fluency and Verbal Short‐Term Memory and a greater change in the PM group for Foot Laterality Recognition. Holm‐corrected significant *p*‐values are indicated with an asterisk.

Abbreviations: CI, confidence interval; MUS, music intervention; PM, psychomotor intervention; SE, standard error.

The MUS group improved in Categorical Fluency (estimated marginal mean = 0.54, SE = 0.26, *p*_Holm_ = 0.037), whereas the PM group declined (mean = –0.76, SE = 0.31, *p*_Holm_ = 0.013). A similar pattern emerged for Verbal Short‐Term Memory, with a trend‐level gain in MUS (0.48, SE = 0.26, *p*_Holm_ = 0.064) and a significant decrease in PM (mean = –0.68, SE = 0.31, *p*_Holm_ = 0.027). In contrast, the PM group showed a relative advantage in Foot Laterality Recognition Accuracy, although neither group exhibited significant within‐group changes (MUS mean = –0.38, SE = 0.27, *p*_Holm_ = 0.17; PM mean = 0.47, SE = 0.31, *p*_Holm_ = 0.12).

Additionally, we examined whether training intensity influenced the above‐reported between‐group differences for Categorical Fluency, Verbal Short‐Term Memory, and Foot Laterality recognition. Controlling for age and site, total time spent on homework in the MUS group over 6 months showed a positive association with verbal short‐term memory scores (Pearson's *r*(7) = 0.72, *p* = 0.03, *N* = 11; Figure 6). For Categorical Fluency (Pearson's *r*(7) = 0.03, *p* = 0.95) and Foot Laterality recognition (Pearson's *r*(4) = 0.18, *p* = 0.74), this association was not significant.

**FIGURE 6:**
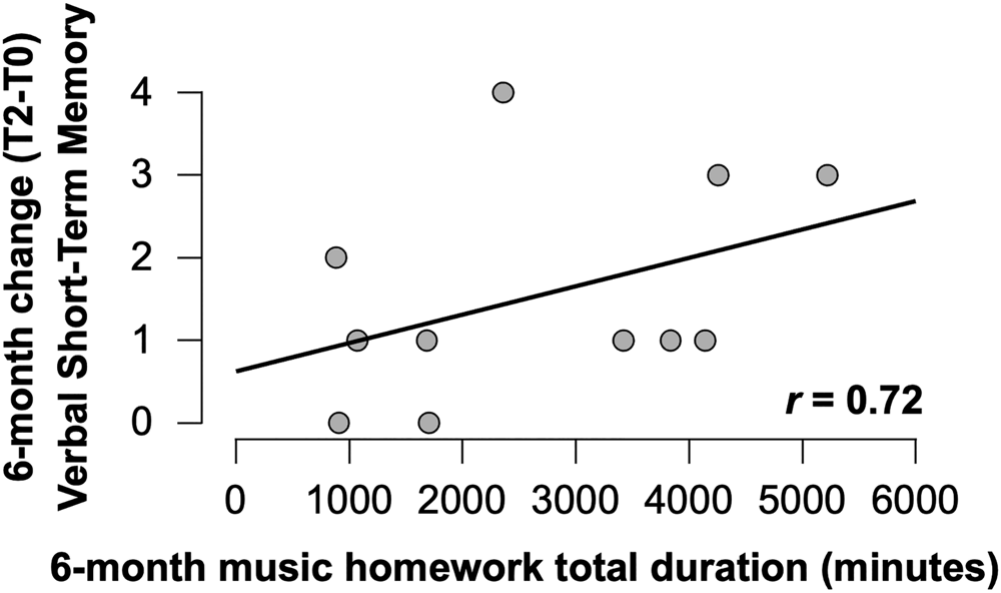
Positive parametric correlation between change in Verbal Short‐Term memory (COGTEL subtest, T2−T0) and total homework duration over 6 months in the MUS group. Abbreviation: MUS, music intervention.

#### 3.5.4 Within‐Group MUS and PM 6‐Month Change (T2−T0) for Sensorimotor and SIN Tasks

Finally, we assessed within‐group change on sensorimotor (PPT and UBT) and SIN tasks. All mean change scores were positive, indicating stable performance without decline. In the PM group, significant gains were found for the PPT Total Score (estimated marginal mean = 6.9, SE = 1.8, *t* = 3.8, *p* = 0.009, Hedges’ *g* = 0.47) and left‐foot balance (mean = 10.3 s, SE = 4.1, *t* = 2.5, *p* = 0.04, Hedges’ *g* = 0.37). The MUS group showed a significant improvement in binaural SRT (mean gain = 0.5 dB, SE = 0.2, *t* = 2.3, *p* < 0.05, Hedges’ *g* = 0.70).

## 4 MRI Outcomes

### 4.1 Hippocampal GM Results (T2−T0)

For left and right GM volume, LMM analyses revealed no significant main effects of Time or Time × Group interactions. A robust Group effect emerged in all models (*p* < 0.001), reflecting consistently greater GM volume in HCs than in MUS and PM participants, who showed evidence of GM atrophy at both time points (*p* < 0.001) and in both hemispheres (left: *F*(1, 33.68) = 12.53, *p* < 0.001, partial *η* ^2^ = 0.271; right: *F*(2, 33.87) = 11.09, *p* < 0.001, partial *η* ^2^ = 0.396; (Figure 7). Within‐group analyses indicated that left/right‐hippocampal GM volume remained stable over time in all groups (HC, MUS, PM; changes were not significantly different from zero).

**FIGURE 7:**
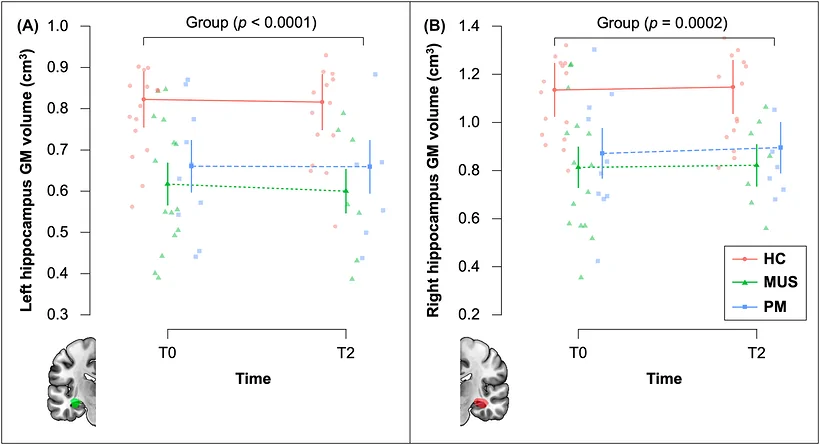
Baseline (T0) and 6‐month (T2) gray matter volume in the left and right hippocampus in MCI participants undergoing MUS (green) or PM (blue) interventions versus HC (red). Significant at *p* < 0.001 uncorrected. (A) Left hippocampal volumes. (B) Right hippocampal volumes. Abbreviations: HCs, healthy controls; MCI, mild cognitive impairment; MUS, music intervention; PM, psychomotor intervention.

### 4.2 Functional Connectivity Results (T2−T0)

As shown in Figure 8A and Table 4, left‐hemisphere network connectivity differed significantly between groups (*F*(2, 35.0) = 7.6, *p* < 0.002, partial *η* ^2^ = 0.303). HCs exhibited higher connectivity than both intervention groups (HC‐MUS: estimated marginal mean = 0.17, SE = 0.04, *z* = 3.7, *p*_Holm_ = 0.0005, Hedges’ *g* = 0.91; HC‐PM: mean = 0.12, SE = 0.04, *z* = 2.9, *p*_Holm_ = 0.008, Hedges’ *g* = 0.64), with no significant MUS‐PM difference (*p*_Holm_ = 0.28). Connectivity remained stable over time in all groups.

**FIGURE 8:**
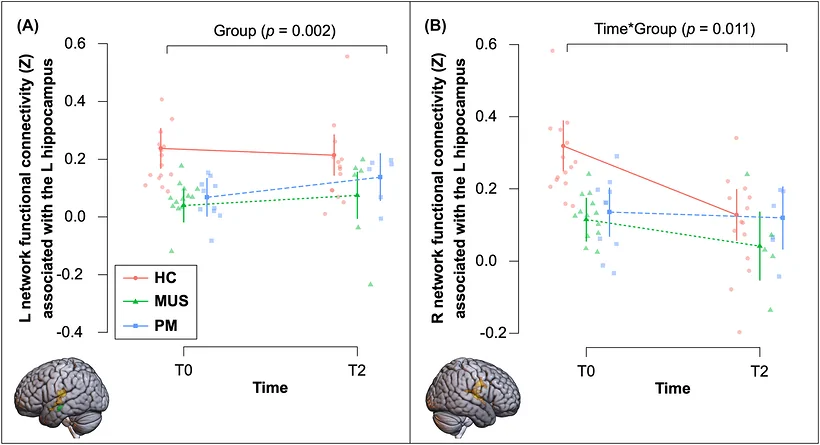
Six‐month longitudinal changes in functional connectivity of left‐hippocampal networks in MCI participants undergoing MUS or PM interventions versus HC. (A) Left network: connectivity remained stable across groups, with HC showing consistently higher values than MUS and PM. (B) Right network: a significant Time × Group interaction, driven by a connectivity decline in HC, whereas MUS and PM groups showed stable connectivity. Significant comparisons are indicated with asterisks. Abbreviations: HCs, healthy controls; L, left; MCI, mild cognitive impairment; MUS, music intervention; PM, psychomotor intervention; R, right.

**TABLE 4:** Estimated marginal mean comparisons (T2−T0) for left‐ and right‐hemispheric hippocampal network functional connectivity.

T2−T0 left hip FC comparison	Estimate	SE	95% CI (lower)	95% CI (upper)	*z*	*p*_Holm_	Hedges’ *g*
HC	−0.02289	0.02800	−0.07685	0.03108	−0.83	0.786	0.205
MUS	0.03544	0.04200	−0.04590	0.11677	0.85	0.786	0.290
PM	0.07021	0.04000	−0.00881	0.14923	1.74	0.245	0.604
**T2−T0 right hip FC comparison**							
HC	−0.19129	0.03312	−0.25621	−0.12638	−5.78	2.296 × 10^−^^8^*	1.445
MUS	−0.07319	0.05023	−0.17165	0.02526	−1.46	0.290	0.501
PM	−0.01608	0.04760	−0.10936	0.07721	−0.34	0.736	0.116

*Note*: Left‐network connectivity remained stable across all groups. In contrast, right‐network connectivity declined significantly only in HC, whereas both intervention groups remained stable. Holm‐corrected significant *p*‐values are indicated with an asterisk. Effect sizes are reported as Hedges’ *g*.

Abbreviations: CI, confidence interval; FC, functional connectivity; HCs, healthy controls; Hip, hippocampus; MUS, music intervention; PM, psychomotor intervention; SE, standard error.

For the right‐hemisphere network, the LMM model revealed significant effects of Time (*F*(1, 33.9) = 13.4, *p* < 0.001, partial *η* ^2^ = 0.28), Group (*F*(2, 37.4) = 5.3, *p* = 0.009, partial *η* ^2^ = 0.21), and their interaction (*F*(2, 33.3) = 5.1, *p* = 0.01, partial *η* ^2^ = 0.24) (Figure 8B). HCs showed a marked decrease in connectivity over time (*p* < 0.0001, Hedges’ *g* = 1.5), whereas in the MUS and PM groups, connectivity remained stable (Table 4).

## 5 Discussion

This 6‐month pilot longitudinal intervention study examined whether music or psychomotor interventions could enhance cognitive, sensorimotor, and neural outcomes in individuals with MCI. Although global cognition, assessed with the COGTEL‐WS, showed no significant group‐level effects, the music group (MUS) demonstrated a marginal increase at 6 months, despite the modest sample size. In contrast, HCs and the psychomotor group (PM) showed no such change. Additional positive domain‐specific outcomes of music and psychomotor interventions further support the potential clinical relevance of these interventions.

### 5.1 Interpreting the Absence of Significant Global Cognitive Change

As a global cognitive composite, the COGTEL‐WS was selected as a conservative benchmark of overall cognition. Given the limited power of this pilot trial and the inherent clinical heterogeneity of the MCI sample, the absence of significant group‐level effects is not unexpected. Consistent with recommendations for early‐phase intervention studies, domain‐specific cognitive measures and neural markers may be more sensitive to subtle or emerging effects [54].

The lack of robust improvement in the COGTEL‐WS may reflect sample size limitations, cognitive heterogeneity in the MCI group, and the challenge of inducing global cognitive change over 6 months. Nevertheless, the directionally positive trend in MUS and convergent domain‐specific improvements in both intervention groups suggest potential functional relevance of the interventions.

Our findings are consistent with prior music‐based randomized controlled trial (RCTs) in MCI patients, including Biasutti and Mangiacotti [27], which informed our study design [52]. While that study reported improvements in MMSE scores after approximately 4 months of music interventions compared to soft gymnastics, the MMSE is primarily a dementia screening tool. In contrast, we employed the COGTEL‐WS [53], which captures a broader range of cognitive domains and remains sensitive in healthy older adults, thus providing a more conservative outcome measure.

Importantly, Biasutti and Mangiacotti [27] observed improvements only in participants with baseline MMSE scores ≤ 23, whereas those with scores ≥ 23 showed no change. Our sample was restricted to early‐stage MCI (MMSE ≥ 23 or MoCA ≥ 17); our findings thus converge with this pattern. Concerning cognitive scores, the final number of participants in the music group in our study and in that of Biasutti and Mangiacotti [27] for MCI with an MMSE ≤ 23 is identical (*n* = 11).

Finally, the intervention in Biasutti and Mangiacotti [27] involved multimodal musical activities with relatively low motor and coordinative demands. In contrast, our intervention required participants to learn a relatively complex instrument (a tongue drum) that involved bimanual coordination across 13 spatially nonlinear notes, consistent with a latency phase typical of early complex skill acquisition.

Prior randomized studies in healthy older adults using comparable music‐based interventions from our group have shown behavioral and neuroplastic changes emerging after approximately 6 months of interventions [16, 29−31], and in the brain functional domain only after 12 months [15].

A larger RCT over more than 6 months in a well‐characterized MCI cohort is needed to determine whether early domain‐specific and neural effects may translate into robust global cognitive benefits.

### 5.2 Complementary Domain‐Specific Cognitive Benefits

The music practice interventions elicited selective enhancements in verbal fluency and verbal short‐term memory. These gains are consistent with the cognitive demands inherent to musical practice and learning, including maintaining rhythmic and melodic sequences in memory and continuously monitoring performance errors. Moreover, the observed association between homework duration and verbal short‐term memory indicates a potential association between practice intensity and this cognitive improvement. These enhancements also align with evidence that semantic fluency is not only vulnerable early in MCI but is among the most sensitive predictors of conversion to dementia [85, 86].

Improvement of verbal fluency following music practice is of particular interest, as it represents a complex cognitive ability. It relies on general executive control (e.g., maintaining task goals and monitoring responses), more specific executive processes (including working memory updating and cognitive flexibility), and lexical knowledge [87].

The psychomotor interventions improved foot‐laterality judgment (reflecting body‐schema awareness), manual dexterity, and whole‐body balance, domains that reflect proprioceptive processing and sensorimotor control. These findings support the relevance of body‐oriented interventions for cognitively and physically fragile older adults, who are likely to experience increasing challenges in coordination and postural stability. This pattern of results is consistent with the broader psychomotor therapy literature, which shows links between balance, body‐schema functions, and global cognition in aging and MCI [88].

### 5.3 Sensorimotor Challenge Reinforces the Value of Nonpharmacological Multimodal Interventions

Both interventions were associated with positive changes or relative stability in sensorimotor domains, including unimanual and bimanual dexterity and coordination, as well as balance, supporting evidence that low‐intensity coordinative activities may help maintain functional mobility [14]. Importantly, hand dexterity and bimanual coordination remained stable or improved in MCI but declined in HCs, suggesting that these changes are unlikely to stem from test−retest effects and may reflect genuine intervention‐related maintenance [74]. Such improvements in dexterity are functionally meaningful, as they relate to autonomy in everyday activities.

#### 5.3.1 Higher‐Order Auditory Functioning

Improvements in SIN performance after music training and practice point to strengthened auditory‐cognitive and auditory‐motor processes, including selective attention, inhibition, working memory, and temporal synchronization, extending findings in typical aging [29]. Because SIN deficits in MCI may lead to poorer communication, social withdrawal, accelerated neurocognitive decline, and increased dementia risk [89], such gains likely reflect more effective top‐down control and greater functional resilience in everyday listening. This interpretation aligns with recent evidence connecting SIN performance with cognitive control and aging trajectories [10].

#### 5.3.2 Hippocampal GM Stability and Differential Functional‐Connectivity Trajectories

First, the behavioral MCI diagnoses were supported by consistently lower GM volumes in both right and left hippocampus in MCI, in both MUS and PM, compared to HC. Second, although hippocampal GM was reduced at baseline in both MCI groups compared to HCs, left and right hippocampal GM volumes remained stable over 6 months in all three groups. Given that hippocampal atrophy typically progresses more rapidly in MCI than in normal aging [90, 91], this relative stability is noteworthy and may be compatible with neural maintenance, although it cannot be specifically attributed to the interventions. Nevertheless, significant hippocampal volume loss has been demonstrated in individuals with MCI over periods as short as 6 months [92].

This interpretation should be considered in light of prior studies on music interventions in healthy older adults comparing piano practice (PP) with musical culture (MC) instruction. These studies showed that training‐related brain changes are not uniformly characterized by structural growth but instead reflect a combination of region‐specific increases, stability, and decline. For example, Worschech et al. [65] reported increased cortical thickness in bilateral auditory cortices in the PP group relative to MC, whereas Jünemann et al. observed stabilization of fornix WM integrity in the PP group alongside decline in controls. Similarly, Marie et al. [16] found localized GM increases in both groups ([16] Figure 3) and stabilization in the right TE1.0 auditory cortex in the PP group compared to decline in MC ([16] Figure 6), while also reporting generalized whole‐brain atrophy, including in the hippocampi, in both groups ([16] Figure 7).

In this context, the present findings in MCI, characterized by hippocampal stability rather than volumetric increase, are more consistent with neural preservation or slowing of disease‐related degeneration than with de novo structural plasticity. Accordingly, maintenance of hippocampal integrity over time may represent a meaningful intervention effect in a clinically vulnerable population. Importantly, the absence of a consistent decline in HCs over this short interval does not contradict this interpretation, as structural changes in normal aging are often subtle and may not be detectable in modest samples, whereas MCI is associated with accelerated hippocampal atrophy, rendering the observed stability particularly noteworthy.

One of the most notable findings was the stabilization of right hippocampal connectivity in both MUS and PM, in contrast to a significant decline in HCs. This suggests that enriched behavioral stimulation, particularly combined cognitive and sensorimotor training, may help counteract functional disconnection in vulnerable networks and attenuate age‐related decline [14]. As functional connectivity alterations often precede structural atrophy, this stabilization may represent an early indicator of neuroprotective effects. This is even more remarkable because HCs showed a decline over a period as short as 6 months. Such short‐term decline in normal aging is consistent with recent longitudinal aging studies and further argues against test−retest artifacts as an explanation for the relative stability observed in the intervention groups. Such rapid attrition has also been shown in a much larger group of healthy older adults for GM volume [16]. Moreover, the pattern parallels age‐related decline in hippocampal connectivity observed in normative cohorts [93, 94], supporting the interpretation that multidomain interventions may contribute to neural maintenance.

#### 5.3.3 Integrative Interpretation of Behavioral and Neural Findings

Across behavioral, structural, and functional neural measures, trained MCI participants showed relative stability or improvement over 6 months, contrasting with declines in HCs. This pattern arose in multidomain cognitive‐sensorimotor performance, manual dexterity (Purdue Pegboard), hippocampal GM, and functional connectivity. Hippocampal GM and functional connectivity remained stable in the intervention groups, despite expectations of a steeper age‐related decline in MCI compared to HCs. This convergence across modalities is unlikely to be fully explained by test–retest effects or regression to the mean and is compatible with a training‐related maintenance interpretation. Such findings support emerging evidence that measurable behavioral and neural decline can occur in healthy older adults over relatively short time intervals [16, 74, 93, 94].

### 5.4 Feasibility and Ecological Value

High course attendance and substantial home practice in both groups support the feasibility of the interventions among participants who completed follow‐up. In addition, no study‐related adverse events were reported, supporting their safety over the 6‐month period. Sustained attendance and home practice may also reflect strong engagement. Importantly, activities such as music‐making and mindful movement can be readily integrated into daily life, potentially facilitating long‐term adherence compared with laboratory‐based cognitive or sensorimotor drills.

Overall, the findings show the potential of physically low‐impact, multimodal cognitive‐sensorimotor interventions in early intervention strategies for MCI. These observations align with the adaptive capacity model, which posits that pairing moderate‐intensity neuromuscular challenges with higher‐order cognitive engagement may best protect against age‐related decline and increase resilience [13, 14]. Larger, fully powered studies are needed to confirm the neural benefits and explore long‐term clinical outcomes of such multimodal nonpharmacological interventions.

#### 5.4.1 Advantage of This RCT

This study implemented one of the most intensive music‐based interventions in MCI to date (45 min, twice weekly for 6 months). Two recent reviews reporting positive effects of music practice on cognition in MCI patients [22, 23] describe mostly shorter, less intensive music practice protocols, except for one study by Doi et al. [95]. Additionally, the direct comparison with a novel psychomotor intervention, previously untested longitudinally in MCI, adds methodological innovation and interpretative value.

#### 5.4.2 Future Perspectives

Building on the present findings, future trials should aim to refine both methodological and clinical endpoints. First, domain‐specific composite scores may provide more sensitive primary outcomes than global cognitive measures in early‐stage MCI when interventions last 6 months or less. Second, longer follow‐up periods will be critical to determine whether the observed maintenance of hippocampal structure and connectivity translates into clinically meaningful outcomes, such as reduced conversion to dementia, preservation of daily functioning, and decreased fall risk. Third, larger samples will be necessary to better distinguish intervention‐related effects from natural interindividual variability. In this context, the present study should be considered as feasibility‐driven and hypothesis‐generating, providing a foundation for future fully powered randomized controlled trials targeting multidomain behavioral and neural outcomes in MCI.

### 5.5 Limitations

The primary limitation of this study is the reduced sample size at the 6‐month follow‐up. Attrition is, to some extent, inherent to working with a frail MCI population; however, the concurrent COVID‐19 pandemic likely exacerbated dropout rates by restricting mobility, increasing health concerns, and disrupting routine care. Independently of these pandemic‐related factors, retention in MCI populations may also be challenged by fatigue associated with the frequency and intensity of interventions [27].

The parsimonious analysis presented in Figure 5 should be considered exploratory. The three outcome measures were selected based partly on a priori hypotheses of greater cognitive effects of music training and greater sensorimotor effects of the psychomotor intervention. However, the individual outcome measures were not prespecified, and their selection was also informed by visual inspection of the data. Although comparisons were adjusted for multiple testing using the Holm procedure, the findings should be regarded primarily as hypothesis‐generating, informing outcome selection and prespecified hypotheses for future large‐scale studies. This is particularly relevant given the limited number of RCTs directly comparing different intervention regimens in individuals with MCI.

Moreover, as analyses were restricted to participants with available data and did not follow a strict intention‐to‐treat approach, attrition may have introduced bias if completers differed systematically from noncompleters.

Another limitation of the study is the use of a cognitively healthy passive control group rather than a group with MCI. However, the HC group was not intended to serve as an equivalent comparator for causal inference; instead, it provided a conservative benchmark for assessing behavioral test–retest effects and age‐related neuronal decline in healthy older adults.

An additional limitation concerns the definition of hippocampal ROIs based on baseline group differences (MCI < HC). While this approach enabled the identification of disease‐relevant regions and increased sensitivity in this pilot study, it may introduce bias and limit generalizability. Future studies with larger samples should employ independent or anatomically defined ROIs and whole‐brain approaches to validate and extend the present findings.

The MCI population was heterogeneous, despite differentiation between amnestic and nonamnestic subtypes, which were evenly distributed across intervention groups. The final post‐intervention sample size was too small to allow meaningful comparisons as a function of subtype.

A further limitation concerns the intervention schedule: twice‐weekly on‐site sessions were burdensome for some participants due to travel and physical demands, potentially reducing recruitment and adherence. Future studies may handle these issues through flexible or hybrid formats (in‐person/remote), transportation support, or lower‐frequency yet equivalently intensive protocols.

## 6 Conclusion

Music and psychomotor interventions were feasible, engaging, and safe and were associated with modest domain‐specific cognitive and sensorimotor benefits in MCI. Hippocampal functional connectivity remained stable over time in both intervention groups but declined in HCs, a preliminary pattern consistent with possible neural maintenance. These findings support the inclusion of pleasant, engaging, and ecologically meaningful group activities in early interventions targeting neurocognitive decline and dementia risk. Given that gains were observed in domains predictive of dementia conversion, such as semantic fluency, SIN perception, and indicators of fall risk, these interventions hold promise for functional stabilization in the early stages of MCI. Given the study's pilot nature, small sample size, and nonequivalent control group, these findings should be considered preliminary and hypothesis‐generating. Larger, fully controlled trials are required to determine the efficacy of the interventions and whether they exert neuroprotective effects.

## Author Contributions

C.E.J.: Principal investigator; grant acquisition; study conceptualization; project supervision; manuscript writing, review, and editing. C.J.T.: Psychomotor intervention program development; study conceptualization; manuscript review. M.K.: Contribution to funding acquisition; study conceptualization; manuscript review. D.K.: MRI and behavioral data acquisition; preliminary data preprocessing and analysis. G.A.: Clinical participant recruitment and support; study conceptualization; manuscript review. G.B.F.: Clinical participant recruitment and support; study conceptualization; manuscript review. A.B.G.: Clinical participant recruitment and support; study conceptualization; manuscript review. D.M.: Co‐investigator; grant acquisition; study conceptualization; investigation; MRI data acquisition; data curation; formal analysis; original manuscript drafting.

## Conflicts of Interest

The authors declare no conflicts of interest.

## Supporting information




**Supplementary Material**: nyas70433‐sup‐0001‐SuppMat.pdf

**Supplementary Material**: nyas70433‐sup‐0002‐SuppMat.pdf
