# Supplementary material for: Six‐Month Music Practice Versus Psychomotor Interventions in Mild Cognitive Impairment: Behavioral and Hippocampal Outcomes
Source: Ann N Y Acad Sci. 2026 Nov 2;1565(1):e70433. doi: 10.1111/nyas.70433 (PMC13726704; doi:10.1111/nyas.70433)
Supplement: Supplementary file 1 [file NYAS-1565-0-s001.pdf]

Supplementary material 2.1 – Tongue drum musical training

Musical training intervention

The steel tongue drums used in the study were modern idiophone percussion instruments measuring approximately 12 inches (30.5 cm), lightweight and easy to handle. Each participant received an instrument, together with a carrying bag, for use throughout the intervention period and for home practice.

The instruments contain 13 semitone intervals, forming a complete chromatic octave plus one additional octave note. They can be played either with the fingers or with mallets. The instruments provide strong sensorimotor feedback, allow melodic and harmonic group playing, and produce a mellow, pleasant sound, particularly suitable for collective music-making. Different musical styles were practiced throughout the intervention ("from Bach to the Beatles").

Figure 1. Playing the 13-tone tongue drum

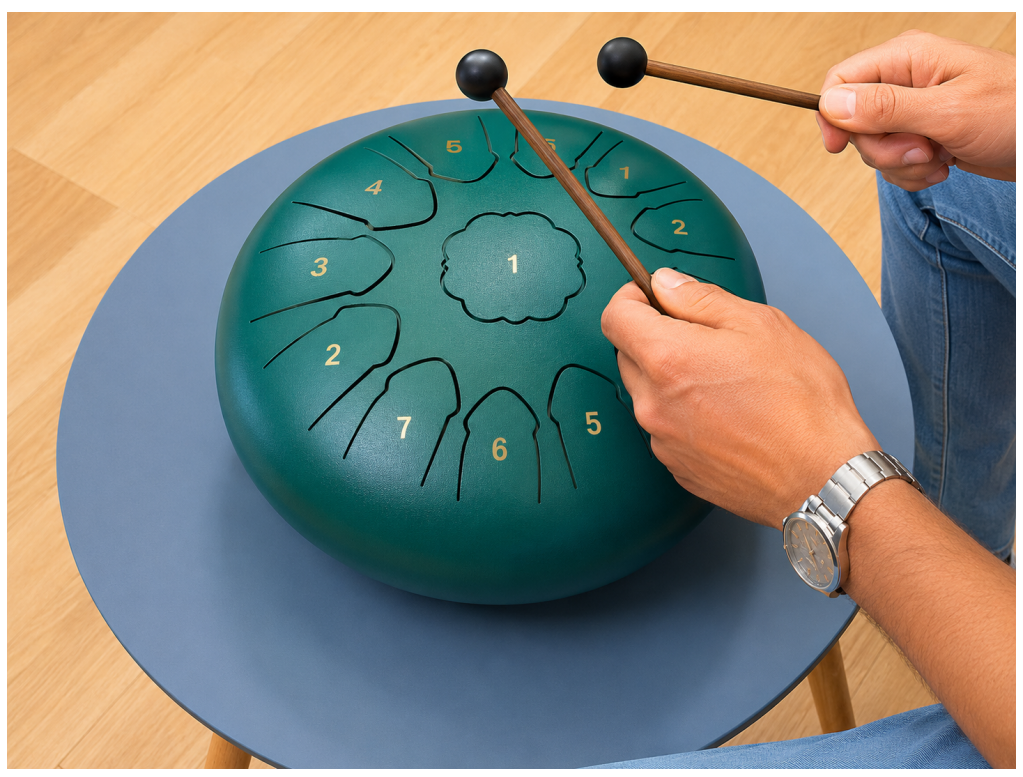

Unlike a piano keyboard, where notes are arranged linearly from left to right, the tongue drum uses a circular and non-linear spatial layout designed for ergonomics and harmonic resonance. Notes are distributed in a balanced zigzag-like configuration, meaning that scales and melodies are learned through movement patterns and spatial memory rather than by visually progressing from left to right (see **Figure 1).**

Training organization

Group instruction, individual coaching, and collective music-making were alternated throughout the 6-month intervention sessions (n=40, 45 minutes each, twice per week). Participants were encouraged to practice daily at home (~20 min/day). Homework included musical exercises and rehearsal of pieces learned during the sessions. To facilitate learning and support memory during early sessions and home practice, a simplified visual notation system based on numbers, colors, and spatial positioning was initially used (see **Figure 2).** Audio recordings were also provided to support home practice. Traditional musical notation was progressively introduced depending on the level of participant group.

Throughout the course, three complementary memory systems were engaged: auditory, visual, and kinesthetic.

Phase 1 – Sensorimotor exploration and familiarization

The first learning phase focused on sensory discovery of the instrument and acquisition of basic playing posture. Participants explored the sound qualities of the tongue drum through free experimentation using fingers and mallets. Attention was directed toward body alignment, grounding, breathing, and avoidance of unnecessary tension in the shoulders, arms, hands, and back. Exercises emphasized controlled arm movements, bilateral coordination, rhythmic alternation between hands, and auditory awareness of resonance and vibration. This exploratory phase aimed to establish a secure sensorimotor foundation before melodic learning.

Phase 2 – Progressive acquisition of scales and spatial-motor references

Participants progressively learned ascending and descending scales and simple rhythmic sequences. Training emphasized the development of spatial and motor landmarks on the circular instrument layout. Visual, auditory, and kinesthetic memory systems were mobilized simultaneously to facilitate note localization and movement sequencing. Simple and familiar melodies were introduced very early in training to promote motivation, musical pleasure, and immediate engagement in active playing. Colors, numbers, note lengths, and simplified visual representations were used to support learning and reduce cognitive load (**Figure 2**).

Figure 2. Simplified visual notation of the opening of *Brother Jacob*. Notes are represented by rectangles of different lengths, colors, and numbers: rectangle length indicates note duration, while color and number indicate pitch. The graphical illustration below represents the instrument note layout using the same color and numerical coding system to show note position and progression on the instrument.

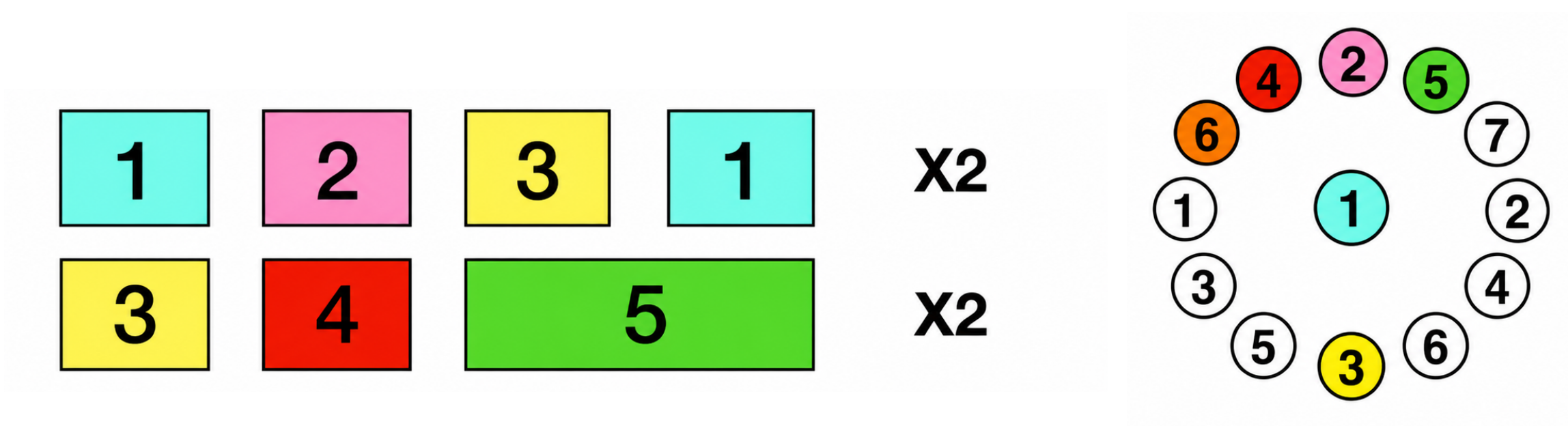

Phase 3 – Transition toward structured musical practice

As training progressed, participants gradually associated the numbered notation system with conventional musical note names and eventually with traditional musical notation (**Figure 3**). Exercises evolved from imitation and guided repetition toward autonomous reading and performance of more complex musical excerpts. Choices of pieces were also adapted to requests and preferences of the participants (**Figure 4**). Participants increasingly practiced multi-voice playing and canons, increasing coordination with other group members. Musical expression and enjoyment progressively became central aspects of the intervention.

Figure 3. *Jingle Bells* (medium difficulty)

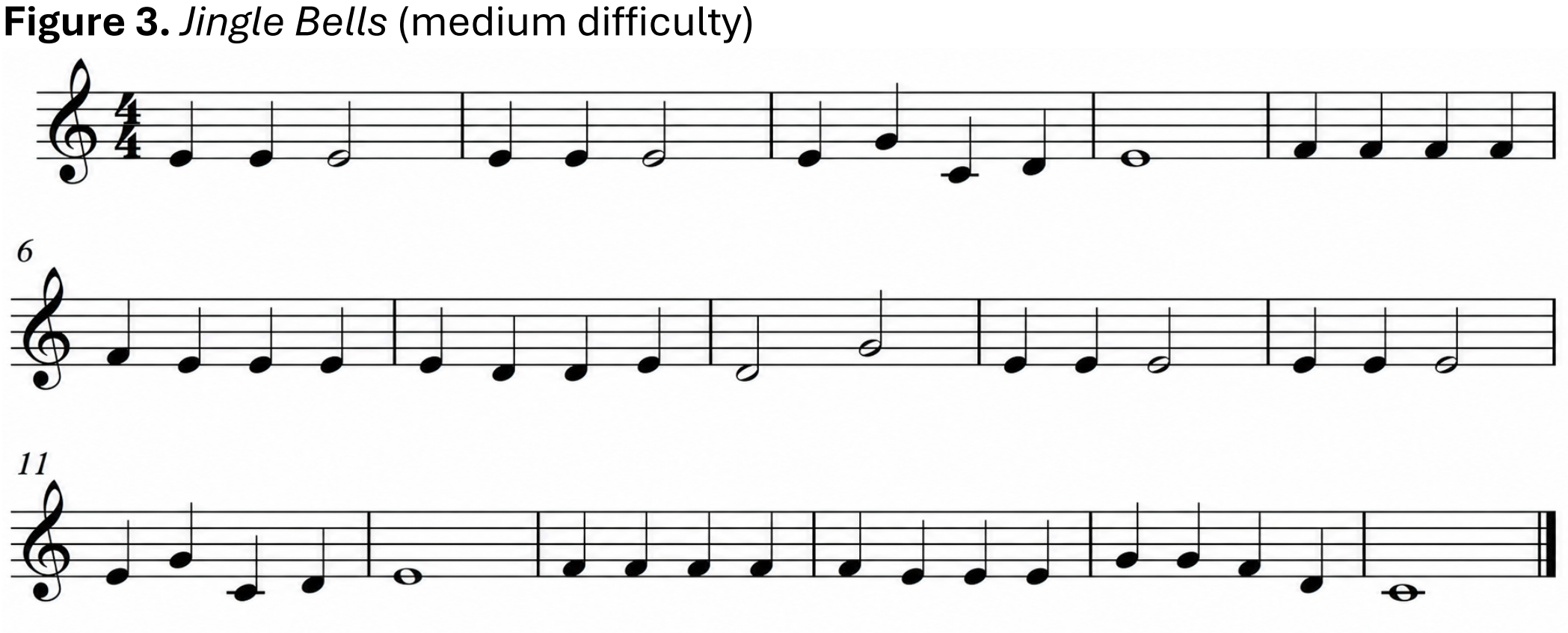

Supplementary material 2.1 – Tongue drum musical training

Figure 4. Piece of choice of the participants: *The Blue Danube* (Johan Strauss junior; advanced level).

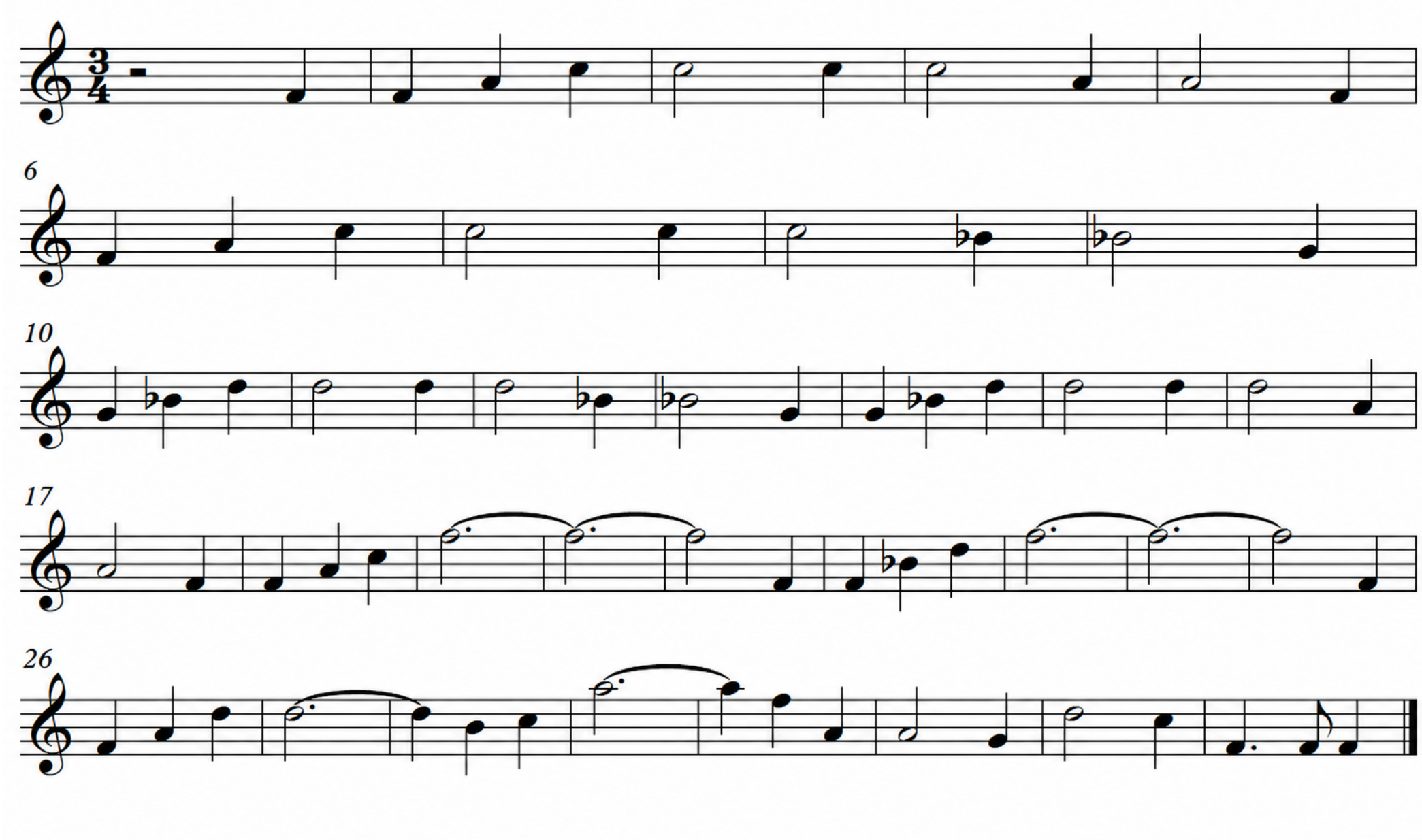

Finally, participants also composed simple scores and practiced structured improvisation based on predefined note sets and accompaniment patterns, providing a basic harmonic and melodic framework that supported creative exploration.

Therapeutic and pedagogical principles

The intervention combined repetition, imitation, auditory feedback, and progressive autonomy. Learning sequences alternated between therapist demonstration, collective performance, individual correction, and peer-supported playing.

Throughout the course, three complementary memory systems were continuously engaged: auditory, visual, and kinesthetic.

The circular arrangement of the instrument encouraged the development of spatial memory and sensorimotor integration, while its pleasant tonal properties reduced fear of mistakes and facilitated improvisation. Across sessions, participants progressively improved rhythmic stability, bimanual coordination, posture control, auditory discrimination, and musical confidence.

The intervention was designed to combine cognitive stimulation, sensorimotor learning, social interaction, and emotional engagement through progressively structured musical practice.

Supplementary material 2.2 – Psychomotor interventions

Psychomotor therapy

Psychomotor therapy sessions gradually enhanced bodily awareness via movement, sensory experiences, and mental representation.

Participants received an instruction booklet with photos like those in the descriptions below to support daily home practice (~20 minutes).

Each of the 6-month intervention sessions (n=40, 45 minutes each, twice per week).
began with a welcome phase, during which participants could share how they felt that day, whether they had practiced at home, as well as their memories and key elements from the previous session.

This was followed by sensory awakening exercises, such as self-massage with the hands or using a ball, or full-body warm-ups, focusing on bodily sensations (temperature, texture, muscle tone, etc.). This approach enabled participants to engage with their bodies holistically and ground themselves in the present, thus preparing them for movement.

Sessions then advanced toward body awareness and expression. Initially focused on walking (balance, grounding, coordination), the program gradually integrated more complex tasks: posture imitation, variations in rhythm and speed, muscle tone modulation, and memory-based activities (movement sequences, coded associations). Use of diverse materials (e.g., balls, weighted objects, fabrics) enriched sensory input and motor engagement, while encouraging creativity.

Sessions concluded with the progressive learning of 12 structured movement sequences inspired by Tai Chi-Chuan (e.g., the Phoenix, the Earth), supporting consolidation and memorization. These sequences required conscious, controlled, fluid movements synchronized with breathing; repetition promoted both relaxation and motor learning. Participants practiced at home and progressively combined sequences.

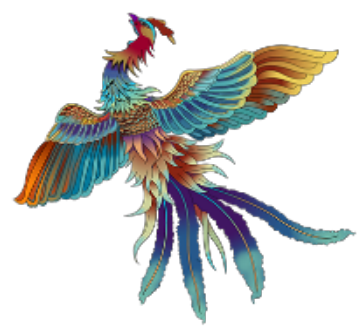

The Phoenix (or the Crane bird):

The Phoenix movement was learned through a standardized framework.

1. A short narrative about the Phoenix, aimed at enabling participants to visualize and mentally represent the bird, and to give meaning to the movement sequence, highlighting the role of imagination and mental representation;
2. Movement learning through imitation, first following precise verbal instructions with eyes open, then with eyes closed, with adjustments provided by the psychomotor therapist;
3. Mental motor imagery, internal visualization followed by alternation between imagery and execution without model or verbal guidance;
4. Verbalization, peer description to reinforce encoding;
5. Session closure

Story

"The Phoenix is a legendary bird. It resembles a massive eagle with its broad magnificent wings, which it opens and closes as it flies through the vastness of the sky. It can live for a very long time because it regenerates itself identically. When it senses its end approaching, it builds a nest of aromatic plants and incense. It sets it on fire and flaps its wings to intensify the flames and consume itself. From the ashes, it is reborn as a young phoenix".

Preparation for the Phoenix movement

This sequence targets upper limb opening and closing with specific hand positioning. Preparatory work includes:
a) elastic band exercises;
b) partner-based tactile feedback to enhance wrist awareness

Supplementary material 2.2 – Psychomotor interventions

Movement learning

See **Figure 1** for the movement guidance provided in the instruction booklet for the Phoenix movement series.

Figure 1. *The Phoenix*

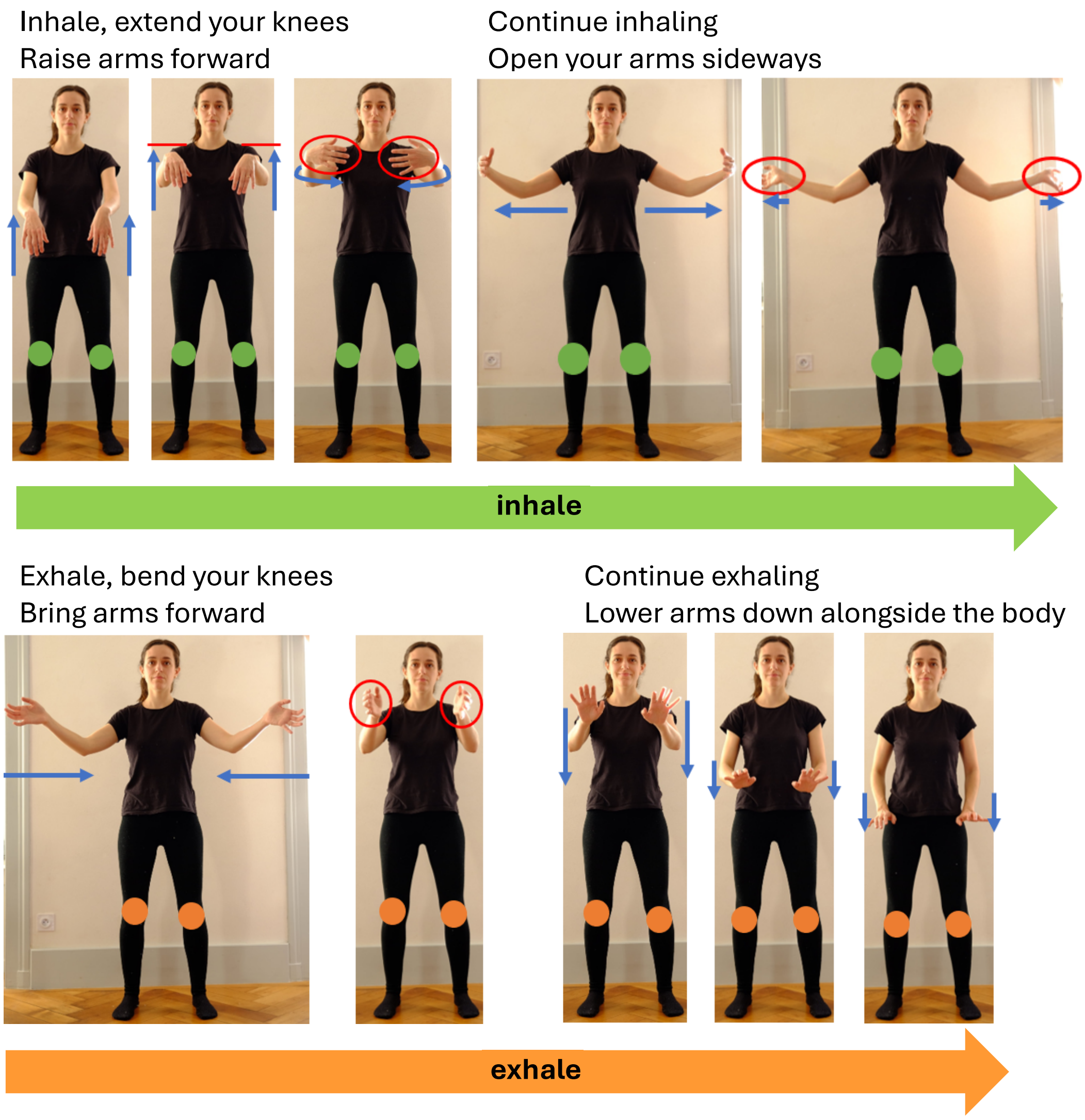

The Earth:

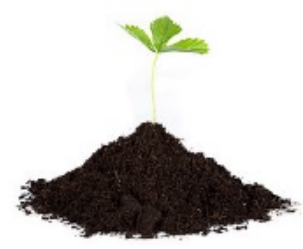

As with the Phoenix movement, the sessions followed the same successive-stage organization described above.

Story

"Spring is now well established, and it is time to take care of the garden. You have bought beautiful shrubs that will bloom around your house. Once you have chosen the right location, you lean toward the ground and scoop up the soil with your hands; it smells of fresh humus. This scent brings back memories of childhood and gradually fills your entire body. Then you take a little soil in your hands, hold it up to the sun to warm it, and let it fall like rain. "

Preparation for the Earth movement

This movement involves lowering the arms during exhalation to gather earth, lifting it upward, and returning it downward, symbolizing a cyclical process. Preparatory exercises included:

a) use of materials (earth, rice, coffee beans) placed at participants' feet;
b) lifting weighted objects;
c) reuse of previously learned "piston" movements;
d) emphasis on circularity (earth returning to earth).

Supplementary material 2.2 – Psychomotor interventions

Movement learning

See **Figure 2 for** the movement guidance for the Earth movement series in the instruction booklet.

Figure 2. *The Earth*

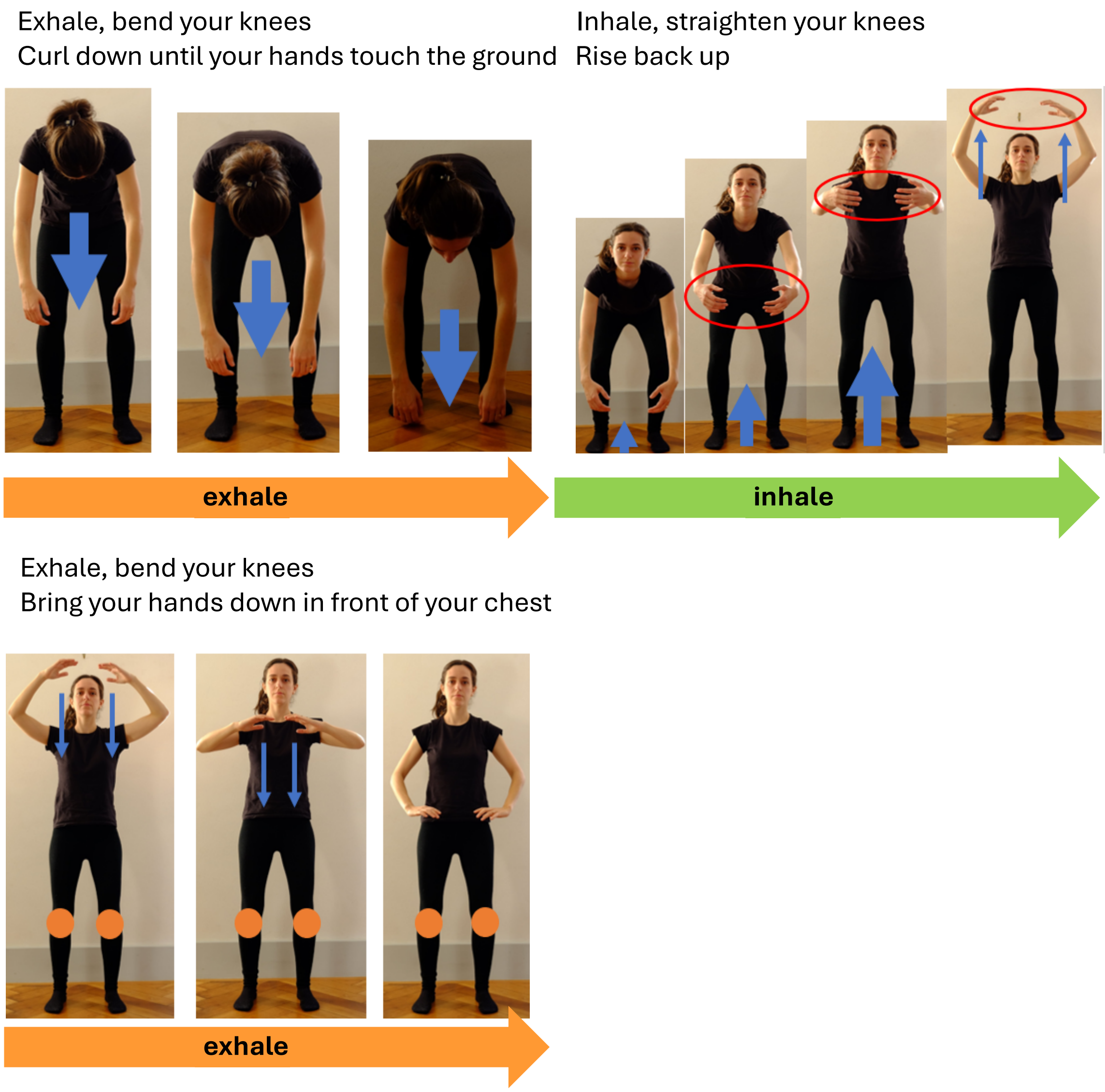

Therapeutic and pedagogical principles

The intervention combined movement imitation, sensory exploration, body awareness, and progressive autonomy through structured psychomotor activities. Sessions alternated between therapist demonstration, guided movement sequences, individual adaptation, partner work, and group interaction. Throughout the course, complementary sensory, cognitive, and motor processes were continuously engaged, including visual, proprioceptive, tactile, and vestibular processing, as well as attentional, body representation, and movement imagery mechanisms.

The intervention was designed to combine cognitive stimulation, sensorimotor learning, emotional engagement, and relational interaction through progressively structured psychomotor practice.
