# Supplementary material for: Six‐Month Music Practice Versus Psychomotor Interventions in Mild Cognitive Impairment: Behavioral and Hippocampal Outcomes
Source: Ann N Y Acad Sci. 2026 Nov 2;1565(1):e70433. doi: 10.1111/nyas.70433 (PMC13726704; doi:10.1111/nyas.70433)
Supplement: Supplementary file 2 [file NYAS-1565-0-s002.pdf]

Supplementary Material 1

Diagnostic Procedures for Mild Cognitive Impairment (MCI)

Overview

MCI diagnoses were established independently at two certified memory clinics in Switzerland:

- Geneva University Hospitals (HUG); Memory Clinic
- Lausanne University Hospital (CHUV; Leenaards Memory Center)

Both centers follow standardized multidisciplinary diagnostic procedures aligned with international recommendations and clinical best practice, including European memory clinic frameworks [1], contemporary intersocietal recommendations for the diagnosis of neurocognitive disorders [2], and the Lausanne Memory Center clinical model [3].

---

1. Geneva Memory Center (HUG)

Diagnostic framework
Diagnosis followed established criteria for Mild Cognitive Impairment [4], within a memory clinic-based multidisciplinary framework [1] and consistent with contemporary European recommendations integrating clinical, cognitive, and neuroimaging information [2].

Assessment procedures

Clinical evaluation
- Medical history
- Neurological examination
- Assessment of subjective cognitive complaints

Cognitive screening
- Mini-Mental State Examination (MMSE)
- and/or Montreal Cognitive Assessment (MoCA)

Paraclinical assessment
- Routine blood tests
- Structural MRI (used to exclude alternative neurological or systemic causes of cognitive impairment)
-

Neuropsychological assessment
- Standardized battery (~1.5 hours)
- Coverage of major domains:
    - Episodic memory
    - Executive functions
    - Attention
    - Language
    - Visuospatial abilities
- Battery harmonized with CHUV procedures

Normative framework and threshold

- Performance interpreted relative to:
    - age-adjusted norms
    - education-adjusted norms
- Impairment defined based on:
    - neuropsychological performance
    - clinical judgment
    - functional status
- Typical range:
    - approximately **1–1.5 SD below normative expectations**, but not applied as a strict cut-off

Diagnostic principle

MCI diagnosis required:

- Objective cognitive impairment in ≥1 domain
- Preserved functional autonomy
- Absence of dementia

---

2. Lausanne Memory Center (CHUV – Leenaards Memory Center)

Diagnostic framework

Diagnosis followed a clinically driven approach emphasizing functional preservation, as detailed in Damian et al. (2018), within a European memory clinic framework (Frisoni et al., 2023) and consistent with intersocietal recommendations emphasizing integrated clinical and functional evaluation (Frisoni et al., 2024).

Core diagnostic principle

MCI defined as:
Cognitive impairment **without loss of functional independence in daily life**

Functional assessment

Operationalized using:

- Clinical Dementia Rating (CDR): 0.5
- Activities of Daily Living (ADL): 6/6
- Instrumental ADL (IADL): 8/8

Cognitive assessment

- Clinical evaluation
- Neuropsychological testing across domains:
    - Memory
    - Executive function
    - Attention
    - Language
    - Visuospatial abilities

Normative framework and threshold

- No strict psychometric cut-off (e.g., fixed 1.5 SD)
- Diagnosis based on:

- integration of cognitive performance
- preserved daily functioning
- clinical expertise

---

3. Harmonization across centers

Although minor procedural differences existed, both centers shared key diagnostic principles:

- Multidomain neuropsychological assessment
- Use of age- and education-adjusted norms
- Clinical integration of cognitive and functional data
- Exclusion of dementia and alternative etiologies
- Alignment with European memory clinic practice (Frisoni et al., 2023) and contemporary recommendations for multimodal diagnosis (Frisoni et al., 2024)

---

4. Summary

Participants were classified as MCI if they met the following criteria:

- Objective cognitive impairment in one or more domains
- Largely preserved functional autonomy
- No diagnosis of dementia
- Clinical confirmation at a specialized memory center

References

1. Frisoni GB, Altomare D, Ribaldi F, Villain N, Brayne C, Mukadam N, Abramowicz M, Barkhof F, Berthier M, Bieler-Aeschlimann M, Blennow K, Brioschi Guevara A, Carrera E, Chételat G, Csajka C, Demonet J-F, Dodich A, Garibotto V, Georges J, Hurst S, Jessen F, Kivipelto M, Llewellyn DJ, McWhirter L, Milne R, Minguillón C, Miniussi C, Molinuevo JL, Nilsson PM, Noyce A, Ranson JM, Grau-Rivera O, Schott JM, Solomon A, Stephen R, van der Flier W, van Duijn C, Vellas B, Visser LNC, Cummings JL, Scheltens P, Ritchie C, Dubois B. Dementia prevention in memory clinics: recommendations from the European task force for brain health services. The Lancet Regional Health – Europe. 2023;26. https://doi.org/10.1016/j.lanepe.2022.100576

2. Frisoni GB, Festari C, Massa F, Cotta Ramusino M, Orini S, Aarsland D, Agosta F, Babiloni C, Borroni B, Cappa SF, Frederiksen KS, Froelich L, Garibotto V, Haliassos A, Jessen F, Kamondi A, Kessels RP, Morbelli SD, O'Brien JT, Otto M, Perret-Liaudet A, Pizzini FB, Vandenbulcke M, Vanninen R, Verhey F, Vernooij MW, Yousry T, Boada Rovira M, Dubois B, Georges J, Hansson O, Ritchie CW, Scheltens P, van der Flier WM, Nobili F. European intersocietal recommendations for the biomarker-based diagnosis of neurocognitive disorders. Lancet Neurol. 2024;23(3):302–12. https://doi.org/10.1016/S1474-4422(23)00447-7

3. Damian D, Rouaud O, Draganski B, Brioschi-Guevara A, Bortolotti M, Laouadi M-H, Demonet J-F. Memory center:The Lausanne model. Clinical and Translational Neuroscience. 2018;2(1):2514183X18773482. https://doi.org/10.1177/2514183x18773482

4. Petersen RC. Mild cognitive impairment as a diagnostic entity. J Intern Med. 2004;256(3):183–94. https://doi.org/10.1111/j.1365-2796.2004.01388.x
